# Diatoms synthesize sterols by inclusion of animal and fungal genes in the plant pathway

**DOI:** 10.1038/s41598-020-60993-5

**Published:** 2020-03-06

**Authors:** Carmela Gallo, Simone Landi, Giuliana d’Ippolito, Genoveffa Nuzzo, Emiliano Manzo, Angela Sardo, Angelo Fontana

**Affiliations:** 1 National Research Council of Italy, Institute of Biomolecular Chemistry, Bio-Organic Chemistry Unit, Via Campi Flegrei 34, 80078 Pozzuoli (Napoli), Italy; 2 Univeristy of Naples “Federico II”, Department of Biology, Cupa Nuova Cintia 21, 80126 Napoli, Italy; 3 Stazione Zoologica “A. Dohrn”, Villa Comunale, 80121 Napoli, Italy

**Keywords:** Biosynthesis, Metabolic pathways, Plant sciences

## Abstract

Diatoms are ubiquitous microalgae that have developed remarkable metabolic plasticity and gene diversification. Here we report the first elucidation of the complete biosynthesis of sterols in the lineage. The study has been carried out on the bloom-forming species *Skeletonema marinoi* and *Cyclotella cryptica* that synthesise an ensemble of sterols with chemotypes of animals (cholesterol and desmosterol), plants (dihydrobrassicasterol and 24-methylene cholesterol), algae (fucosterol) and marine invertebrates (clionasterol). In both species, sterols derive from mevalonate through cyclization of squalene to cycloartenol by cycloartenol synthase. The pathway anticipates synthesis of cholesterol by enzymes of the phytosterol route in plants, as recently reported in Solanaceae. Major divergences stem from reduction of Δ24(28) and Δ24(25) double bonds which, in diatoms, are apparently dependent on sterol reductases of fungi, algae and animals. Phylogenetic comparison revealed a good level of similarity between the sterol biosynthetic genes of *S. marinoi* and *C. cryptica* with those in the genomes of the other diatoms sequenced so far.

## Introduction

Sterols are vital components of all eukaryotic cells where they modulate structure and function of membranes and, as precursors of signaling molecules, they control growth and development. Plant sterols, commonly named phytosterols, are involved in morphogenesis, development, reproduction and stress response^1–3^. Analogously, microalgal sterols showed critical physiological roles related to photosynthesis, growth, light response and fatty acid metabolism^4^.

Sterols are terpenes deriving from a complex process of polymerization of six isoprene units. In animals and fungi, the mevalonic acid (MVA) pathway is the only route for the biosynthesis of the isoprene units isopentenyl pyrophosphate (IPP) and dimethylallyl pyrophosphate (DMAPP). In higher plants and algae, IPP and DMAPP derive either from the MVA pathway in the cytoplasm or the methylerythritol phosphate (MEP) pathway in the plastids^5^. Without a clear cellular compartmentalization, both biochemical routes have been also reported in mosses and streptomyces. Sterol biosynthesis from IPP and DMAPP is generally taxa-specific and proceeds via lanosterol by lanosterol synthase (LSS) in nonphotosynthetic organisms (e.g. animals and fungi) or cycloartenol by cycloartenol synthase (CAS) in photosynthetic lineages (e.g. plants and algae)^6^. Cholesterol is the major animal sterol but its biosynthesis in tomato has been recently shown to derive from a cycloartenol-dependent pathway composed of enzymes either shared with phytosterols or evolved from phytosterol biosynthetic genes^7^. Furthermore, insects and lower eukaryotes, including marine invertebrates and a few microalgae, are suggested to convert phytosterols to cholesterol and *vice versa*^8–11^.

Diatoms represent an important component of the aquatic ecosystem^12,13^ and form the largest biological group of marine phytoplankton^14,15^. These microalgae are major global producers by contributing to fixation of CO_2_ and geochemical cycles in the world oceans^16,17^. Furthermore, they can biosynthesize a number of secondary metabolites^18^ including domoic acid^19,20^, oxylipins^21–24^, carotenoids^25,26^, as well as organic and inorganic biopolimers such as chitin, silica and calcite^27–30^. Recently, we showed that a specific class of sterol derivatives, namely sterol sulfates (StS), operate as chemical trigger of cell death programs in the diatom *Skeletonema marinoi*^31^. The report is new but underlines the possible role of sterols to control cell survival during phytoplankton blooms. Diatoms possess both plant (e.g. brassicasterol) and animal (e.g. cholesterol) sterols^32^. To explain this unconventional blend, Fabris *et al*. have recently suggested a mixed plant and fungal pathway on the basis of molecular studies in the diatom *Phaeodactylum tricornutum*^33^. However, diatoms usually do not produce ergosterol, the typical fungal sterol, as well as they lack homologs of the eukaryotic proteins required for its synthesis from lanosterol^34^. On the whole, the “fungal” hypothesis does not have so far biochemical confirmation and sterol biogenesis is still an undisclosed question in these microalgae.

In the present study, we have investigated the biosynthesis of sterols, from acetyl-CoA to the final products, with the aim to find a unified pathway suitable to produce both plant and animal sterols in diatoms. Combination of transcriptomic approach with labelling methods and GC-MS profiling has been used to disclose the origin of the complex mixtures of sterols in the diatoms *Skeletonema marinoi* and *Cyclotella cryptica*. Post analysis of the molecular data, sterol profiling and comparison with other diatom species provides general significance to this study.

## Results

### Profiling and identification of diatom sterols

**Figure 1:**
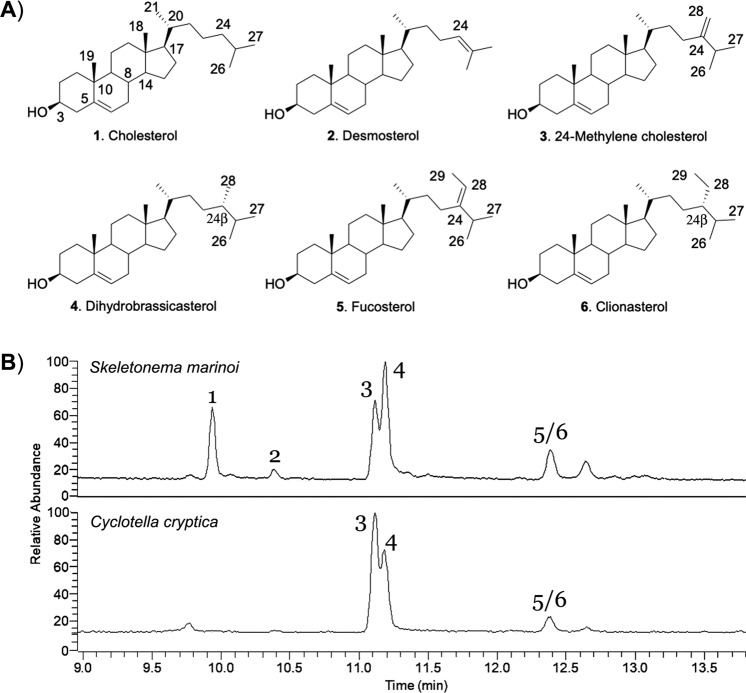
Sterols in *S. marinoi* and *C. cryptica*. (**A**) Structures and (**B**) GC-MS chromatograms of acetylated derivatives. β-chiral descriptor in side chain is in agreement with Nes^6^.

Four different Δ5-sterols, namely 24-methylene cholesterol, dihydrobrassicasterol, clionasterol (also named γ-sitosterol) and fucosterol were identified by standard comparison in the GC-MS profiles of *C. cryptica* (Fig. 1). The above phytosterols together with cholesterol and minor level of desmosterol were also observed in *S. marinoi*. Dihydrobrassicasterol (β-orientation of the methyl group at C-24) was distinguished from campesterol (α-orientation at C-24) on the basis of the ^1^H- and ^13^C-chemical shift of Me-28^35,36^ after purification of the natural product. Analogously, NMR comparison with an original standard confirmed the *E* configuration of fucosterol^37–39^ while stereochemistry at C-24 of clionasterol (β-orientaion of the ethyl group) required a complete NMR analysis after purification by HPLC (Supporting Material). The proton chemical shifts of this natural product were very similar to the values reported in the literature for clionasterol^35,40^ but showed differences with a standard of β-sitosterol for H_3_-26 (0.8308 vs 0.8371 ppm), H_3_-27 (0.8106 vs 0.8163 ppm) and H_3_-29 (0.8578 vs 0.8462) (Supplementary Fig. S1). As shown in Table 1, ^13^C NMR data of the diatom product provided additional agreement with the literature^35^ and corroborated the differences between the two 24-ethyl epimers at C-23 (26.4 vs 26.1 ppm), C-24 (46.1 vs 45.8), C-26 (19.0 vs 19.8) and C-27 (19.6 vs 19.1), thus giving definitive confirmation of the assignment of clionasterol in the diatom extracts.

**Table 1:** ^13^C NMR (CDCl_3_, 125 MHz) data of clionasterol (24β-ethyl epimer) from *C. cryptica* and an authentic standard of β-sitosterol (24α-ethyl epimer).

C	Clionasterol^a^	β-Sitosterol	C	Clionasterol^a^	β-Sitosterol
1	37.3	37.3	16	28.2	28.2
2	31.7	31.6	17	56.0	56.0
3	71.8	71.7	18	11.9	11.8
4	42.3^b^	42.2^c^	19	19.4	19.4
5	140.8	140.7	20	36.3	36.1
6	121.7	121.7	21	18.8	18.8
7	31.9^d^	31.9^d^	22	33.9	33.9
8	31.9^d^	31.9^d^	23	26.4	26.1
9	50.1	50.1	24	46.1	45.8
10	36.4	36.5	25	28.9	29.2
11	21.1	21.0	26	19.0	19.8
12	39.8	39.8	27	19.6	19.1
13	42.3^b^	42.3^c^	28	23.0	23.0
14	56.8	56.8	29	12.3	12.0
15	24.3	24.3			

^a^Natural product; ^b,c,d,e^Overlapped signals.

To provide general significance to these results, we also performed additional GC-MS profiling of the diatoms *Skeletonema costatum*, *Thalassiosira weissflogii*, *Phaeodactylum tricornutum, Pseudonitzschia arenysensis*. In agreement with the most recent reports, 24-methylene cholesterol was the recurrent sterol in these diatoms, often accompanied by dihydrobrassicasterol and cholesterol (Supplementary Table S1). In addition, desmosterol and 24-ethyl cholesterol (likely clionasterol) were found in *P. tricornutum* while desmosterol was present in *S. costatum* and *T. weissflogii*. In agreement with the literature^32,41–44^, the results show an evident variability of the sterol composition in these diatoms but confirm the original assumption that diatoms can in principle biosynthesize sterols from cyclization of both cycloartenol and lanosterol. Another characteristic of the analyzed diatoms is the β configuration at C-24 of dihydrobrassicasterol and clionasterol which is commonly found in organisms, such as algae o fungi, that are considered lower than vascular plants in the evolutionary hierarchy^45,46^.

### Isoprene origin from mevalonate pathway

**Figure 2:**
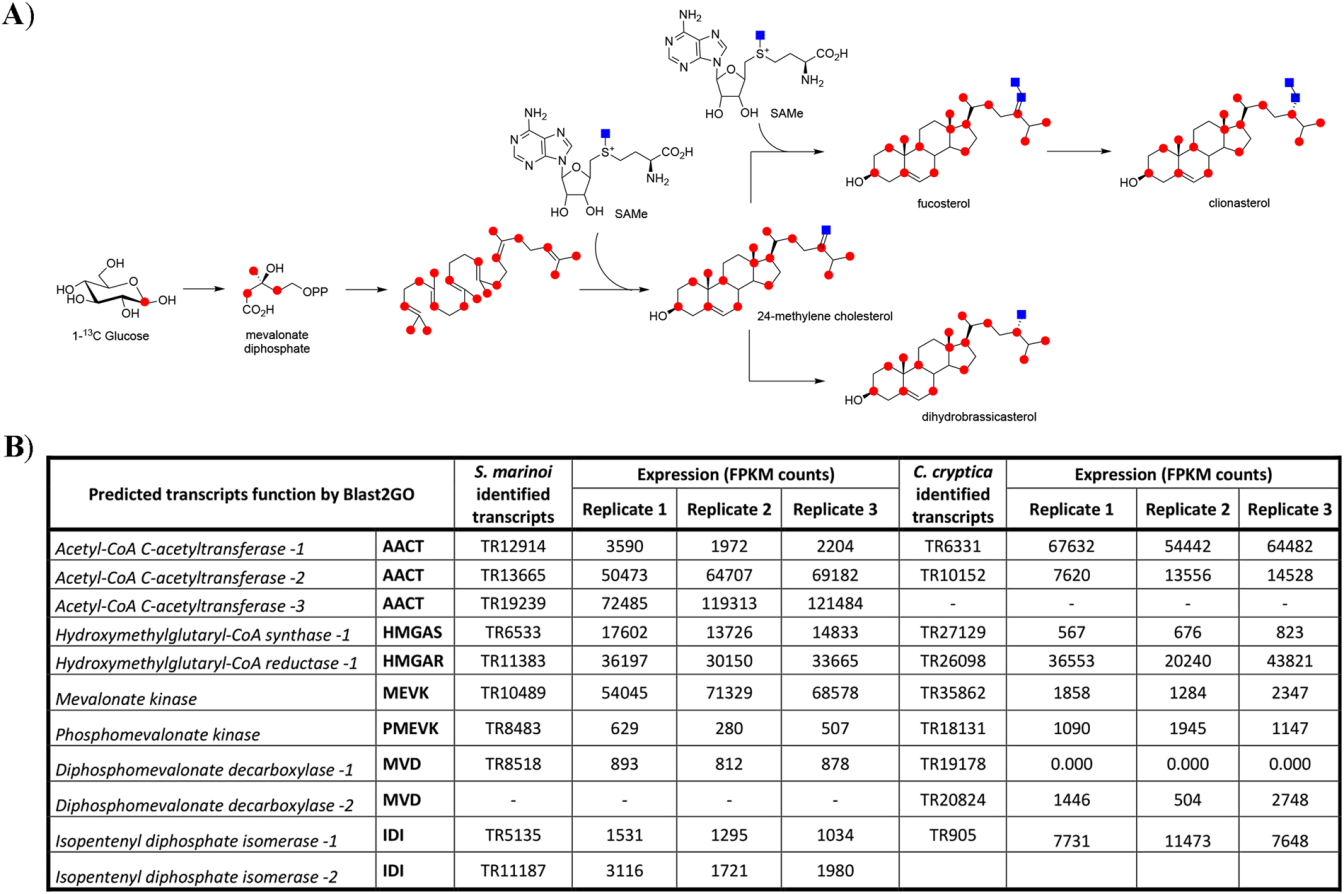
Mevalonate biosynthesis of sterols in *S.marinoi* and *C. cryptica*. (**A**) Incorporation of ^13^C-labelling (red spot and blue square) in dihydrobrassicasterol, fucosterol and clionasterol after feeding of 1-^13^C-glucose to *C. cryptica* under heterotrophic conditions. SAMe = S-adenosyl methionine; (**B**) Transcript sequences of genes coding for enzymes of the mevalonate pathway in *S. marinoi* and *C. cryptica*.

In order to clarify the sterol biosynthetic pathway of *S. marinoi* and *C. cryptica*, transcriptome assembly and sequence alignments were performed by triplicates of each species. The analysis revealed the whole set of genes of the MVA pathway from acetyl-CoA C-acetyltransferase to isopentenyl diphosphate isomerase (Fig. 2). Experimental support to these results was addressed by labelling studies with [1-^13^C]-glucose. Diatoms are photo-autotrophic organisms thus use of an organic substrate required acclimation of the species to heterotrophic conditions. To this aim, *C. cryptica* was grown heterotrophically as previously reported^47,48^. After extraction and fractionation of the cell pellets, labeled and unlabeled dihydrobrassicasterol were compared by ^13^C NMR spectroscopy^36^. Spectra of these products clearly revealed selective incorporation at C-1, C-3, C-5, C-7, C-9, C-13, C-15, C-17, C-18, C-19, C-21, C-22, C-24, C-26, C-27 and C-28 (Supplementary Fig. S2). A few positions such as C-6 (121.74 ppm) and C-16 (28.25 ppm) were not labeled while other carbon atoms (C-1, 37.3 ppm; C-7, 31.9 ppm; C-18, 11.9 ppm; C-19, 19.4 ppm) resulted highly enriched. Mapping of these positions permitted to conclude that labeling was specifically present at C-2, C-4 and C-5 of the six isoprene units that give origin to the rearranged terpene skeleton of dihydrobrassicasterol (Fig. 2). On the contrary, very low or no labeling were observed at carbon positions derived from C-1 of the isoprene units. According to the Rohmer’s method^49^, this enrichment study proved the synthesis of dihydrobrassicasterol unambiguously by the mevalonate pathway with no contribution from MEP. Similar results were obtained for fucosterol and clionasterol (Supplementary Figs. S3 and S4). Intense flanking doublets due to incorporation in vicinal carbons were very evident in those signals, namely C-13/C-18 and C-13/C-17, whose proximity is in agreement with the cyclization of the sterol skeleton from squalene. A strong coupling between C-24/C-28 supported labelling of these carbon atoms in dihydrobrassicasterol. Analogous effects were also observed between C-24/C-28 and C-28/C-29 in fucosterol and clionasterol. The carbon atoms C-28 and C-29 of these metabolites do not originate from the isoprene unit but derive from labelled S-adenosyl methionine (SAM) (Fig. 2). In plants, the two reactions are dependent on sterol 24C-methyl transferase 1 (SMT1) and sterol 24C-methyl transferase 2 (SMT2) that catalyze transfer of the methyl group from SAM to cycloartenol or 24-methylenelophenol, respectively. Orthologs of the sequences related to genes of the MVA pathway in *S. marinoi* and *C. cryptica* were also identified in the published genomes of *Thalassiosira oceanica, Thalassiosira pseudonana, Phaeodactylum tricornutum, and Fragilariopsis cylindrus* (Supplementary Table S2).

### Cyclization of sterol skeleton by cycloartenol synthase

**Figure 3:**
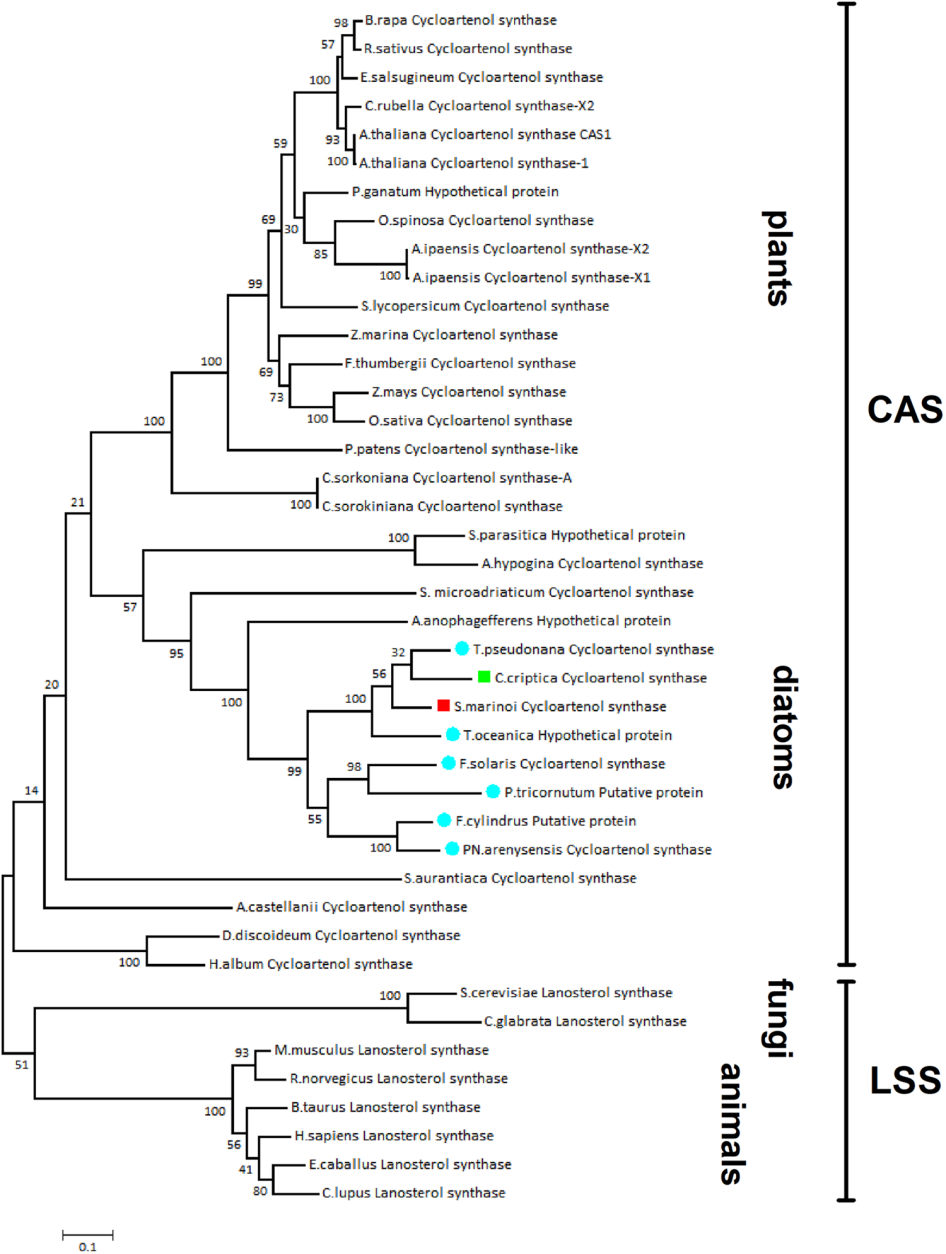
Un-rooted phylogenetic tree of cycloartenol synthase (CAS) and lanosterol synthase (LSS) in diatoms, plants, algae fungi and animals. Alignment used maximum likelihood method. The bootstrapping test (replicate = 100) is reported on each node. Red square = *S. marinoi*; Green square = *C. cryptica*; Light blue spot = other diatom.

Table 2 reports the list of transcripts coding for putative enzymes related to synthesis of sterols from IPP and DMAPP in *S. marinoi* and *C. cryptica*. FPKM counts were obtained for the gene toolbox that oversees each biosynthetic step except for squalene epoxidase (SQE) that did not give confident coverage with any known sequence. Despite the conservation of this enzyme in animals, fungi and plants, more than one polyphyletic group of eukaryotes have shown the lack of the corresponding gene. Very recently, an alternative SQE (AltSQE) belonging to the fatty acid hydroxylase family has been described in diatoms^50^. Analysis of the transcripts of *S. marinoi* and *C. cryptica* supported the presence of orthologs of AltSQE (Sm-TR7561 and Cc-TR29442) thus suggesting that this enzyme catalyzes epoxidation of squalene also in these species. It is worth noting that AltSQE of *S. marinoi* and *C. cryptica*, as well those of other diatoms, showed high sequence similarities (above 60%) with plant Δ5-sterol reductase (DWARF7) that is responsible for one of the latest steps of the phytosterol biosynthesis. The successive reaction of cyclization is proposed to give cycloartenol by cycloartenol synthase (CAS) that was significantly upregulated in both *S. marinoi* and *C. cryptica*. Orthologous CAS genes were also identified in the published genomes of *T. oceanica, T. pseudonana, P. tricornutum and F. cylindrus*. The phylogenetic analysis of these sequences showed clusterization of these proteins with plant CASs despite the diatom sequences form an independent group from plants (Fig. 3). Within the lineage, the putative enzymes were splitted in two sub-branches with separation of the centric species *S. marinoi, C. cryptica, T. pseudonana* and *T. oceanica* from the pennates *P. tricornutum, P. arenysensis, Fragilariopsis solaris* and *F. cylindrus*. Cyclase activity of *Arabidopsis* CAS1 is strictly dependent on the conserved domains KMQGYNGSQ (406 aa – 414 aa) and TADHGWPISDC (474 aa – 484 aa), with Tyr410, His477 and Ile481 that are key residues for the catalytic mechanism^51^. Putative CAS from *S. marinoi* and *C. cryptica* showed domains [(830 aa - 838 aa) and (910 aa- 920 aa) in *S. marinoi*; (353 aa - 361 aa) and (436 aa - 446 aa) in *C. crytica*] and catalytic residues (Tyr834, His913, Ile917 in *S. marinoi*; Tyr357, His439 and Ile443 in *C. crytica*) that were identical to those of *A. thaliana* (Supplementary Fig. S5).

**Table 2:** Transcript sequences of genes coding for enzymes of sterol biosynthetic pathway in *S. marinoi* and *C. cryptica*.

Biosynthetic Function	Predicted enzymatic function by Blast2GO		*S. marinoi* transcripts	Expression (FPKM counts)			*C. cryptica* transcripts	Expression (FPKM counts)		
				Replicate 1	Replicate 2	Replicate 3		Replicate 1	Replicate 2	Replicate 3
Terpene elongation and cyclization	*Farnesyl pyrophosphate synthetase*	**FPPS**	TR5774	747	1469	2779	TR24671	1580	1363	1796
	*Squalene synthase*	**SQS**	TR5837	7653	9077	10903	TR905	7731	11473	7648
	*Alternative Squalene Epoxidase*	**AltSQE**	TR7561	7708	1005	1765	TR29442	13916	16726	10147
	*Cycloartenol synthase*	**CAS**	TR12960	4902	5597	5266	TR3126	2825	3011	3105
Construction of the sterol skeleton	*Cycloartenol-C-24-methyltransferase -1*	**SMT1**	TR612	399669	547431	495121	TR44522	4872	4562	10006
	*Cycloartenol-C-24-methyltransferase -2*	**SMT1**	—	—	—	—	TR45147	34639	25720	33901
	*Sterol Methyl transferase*	**SMT2**	TR11812	19023	13794	15818	TR29102	66731	62025	70507
	*Sterol Methyl transferase*	**SMT2**	TR21333	24344	13997	14326	TR17021	33805	42406	37980
	*C-4 Methylsterol oxidase*	**3βHSD-D**	TR28093	2287	2523	1960	—	—	—	—
	*Sterol-4-methyl oxidase 1*	**SMO1**	TR11964	40534	37090	35927	TR32604	20979	29827	25702
	*Sterol-4-methyl oxidase 1*	**SMO1**	TR30213	3061	3258	2926	TR28329	0.000	1731	2017
	*Sterol-4-methyl oxidase 2*	**SMO2**	TR319	17019	18736	18676	TR32604	20979	29827	25702
	*Sterol-4-alpha-carboxylate 3-dehydrogenase*	**ERG26/D4C**	TR28078	48329	29541	39087	TR14638	11001	7800	11499
	*Cycloeucalenol cycloisomerase*	**CPI**	TR11937	8673	8864	8943	TR30071	3081	3643	4002
	*Sterol 14 demethylase -1*	**CYP**	TR7602	5822	3161	3628	TR12199	32581	38398	40078
	*Sterol 14 demethylase -2*	**CYP**	—	—	—	—	TR23734	6396	4710	5582
	*Sterol 14 demethylase -3*	**CYP**	—	—	—	—	TR30096	70903	65490	74001
	*Delta 14 sterol reductase*	**C14SR**	TR13312	5630	5771	5383	TR19404	47799	46207	50117
	*Delta 5 Sterol desaturase*	**Δ5 SD/DWARF7**	TR28093	2287	2523	1960	TR48724	0.000	7168	2531
	*7 Dehydrocholesterol reductase*	**Δ7SR/DWARF5**	TR2636	7653	17187	13127	TR24068	10801	9765	9443
	*Delta(24(24(1*)*))-sterol reductase*	**24SR/ERG4**	TR213	14750	18308	18373	TR29411	88345	78049	66050
	*24 dehydrocholesterol reductase -2*	**24-DHCR**	TR10998	45299	64582	64882	—	—	—	—
	*22 sterol desaturase*	**ERG5**	—	—	—	—	TR3228	11535	12460	13684

### C4-Demetylation of the triterpene precursor

C4-demethylation is a crucial step in sterol biosynthesis in plants, mammals and fungi. The biochemical transformation requires the consecutive action of three enzymes widely conserved across phyla, namely sterol-4-methyl oxidase (SMO), 3-hydroxysteroid dehydrogenase/C4-decarboxylase (C4D), and a sterone ketoreductase (SKR)^52^. The complex is tethered to the membrane by ergosterol biosynthetic protein 28 (ERG28), a fourth protein that has no catalytic function but plays a key role in preventing accumulation of biosynthetic 4-methyl sterol intermediates^53,54^. Removal of both methyl groups occurs successively by a single SMO in mammals and yeast, whereas in plants the process proceeds in two steps under control of two different SMOs, named SMO1 and SMO2. The analysis of the transcripts of *S. marinoi* and *C. cryptica* did not reveal any presence of ERG28. In analogy with phytosterol biosynthesis, we found sequences that correlate with plant SMO1 (Sm-TR11964, Sm-TR30213) and SMO2 (Sm-TR319) in *S. marinoi*, whereas the transcriptome of *C. cryptica* showed only one sequence for a putative SMO1 (Cc-TR32604). It is interesting that the BLAST analysis of the putative SMO1 encoded by Sm-TR11964 and Cc-TR32604 indicated a good homology with the human C4 methyl sterol oxidase that participates in the C4-demethylation of cholesterol precursors (Supplementary Table S3). Both diatoms also revealed sequences related to fungal ERG26, a decarboxylating enzyme of the C4D family that is responsible for the formation of 3 oxo-sterols from several 3β-4-carboxysterols in fungi, plants and mammals.

### Biosynthesis of phytosterols

**Figure 4:**
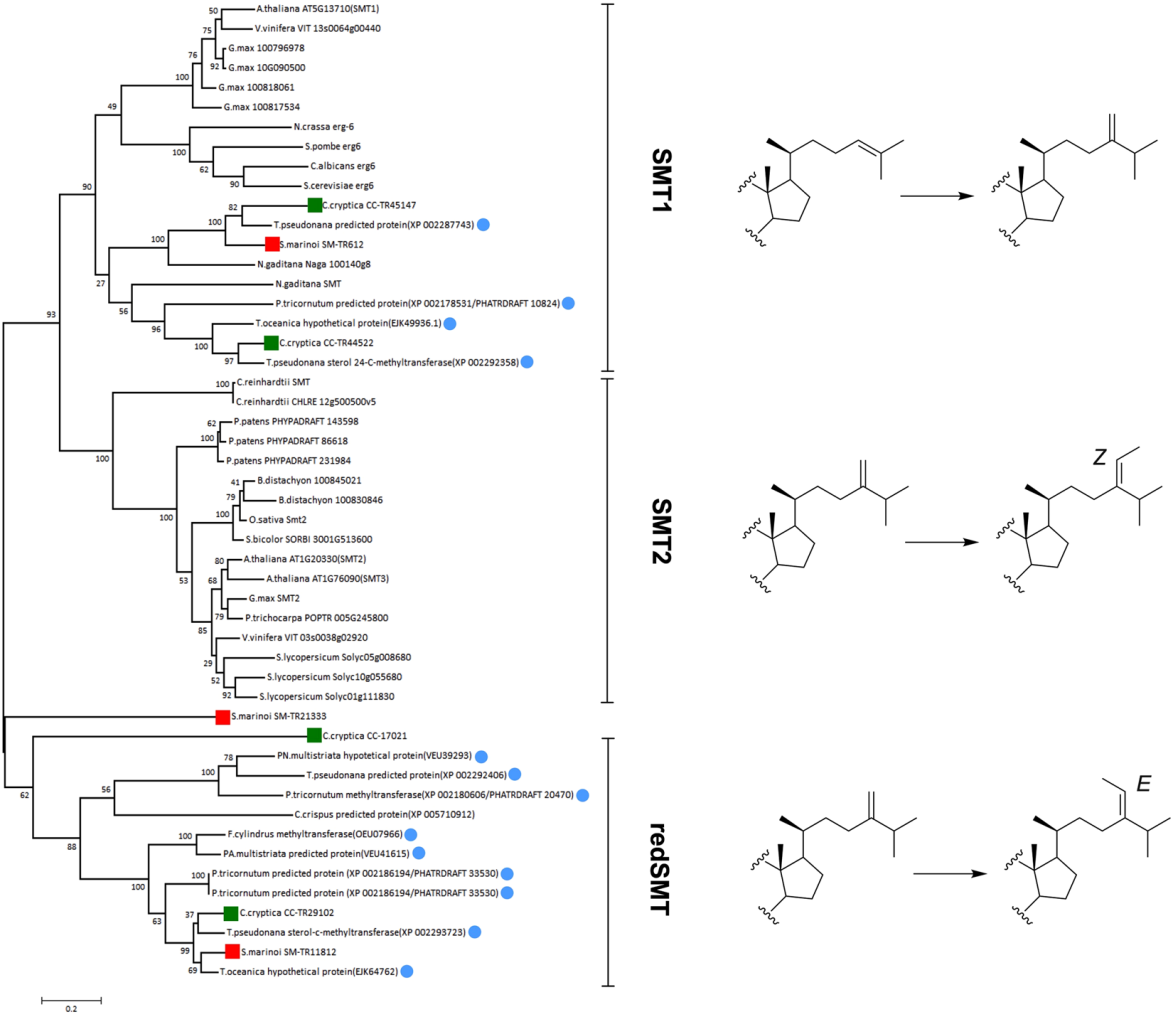
Un-rooted phylogenetic tree of sterol methyl transferase (SMT) of diatoms (Red square = *S. marinoi*; Green square = *C. cryptica*; Light blue spot = other diatom), plants and algae. Reactions catalyzed by SMT are shown next to each sub-family (SMT1, SMT2 and red-SMT). Alignment used maximum likelihood method. The bootstrapping test (replicate = 100) is reported on each node. SMT1 = sterol methyl transferase 1; SMT2 = sterol methyl transferase 2; redSMT = sterol methyl transferase of diatoms and red algae.

*S. marinoi* and *C. cryptica* transcriptomes exhibited respectively, 13 and 16 expressed genes related to the steps leading to C_28_ and C_29_ phytosterols from cycloartenol in agreement with a common plant pathway (Table 2). Expressed genes of key steps in phytosterol biosynthesis showed perfect matching among *S. marinoi* and *C. cryptica*, and high percentage of query coverage and identity with plant genes (Supplementary Table S3 and Supporting Material). Following the labelling results reported above, the analysis revealed orthologs of plant SMT-1 and SMT-2 that are implied in the methylation of cycloartenol and 24-methylenelophenol to give C24-methyl and C24-ethyl derivatives, respectively. On phylogenetic basis, Sm-TR612, Cc-TR44522 and Cc-TR45147 were identified as SMT1 orthologs and clustered with sequences of plants, fungi and other diatoms (Fig. 4). On the other hand, Sm-TR11812, Sm-TR21333, Cc-TR29102 and Cc-TR17021 formed an independent group of SMT that diverged from SMT2 of plants and the green alga *Chlamydomonas reinhardtii* but showed homology with sequences of diatoms and the red alga *Chondrus crispus*. Phylogenesis using the plant nicotinate methyltransferase (NANMT)^55^ as outgroup revealed that differentation of this group of genes from SMT of plants and green algae may have occurred very early and prior to specialization between SMT 1 and SMT2 (Supplementary Fig. S6). We have not proved the catalytic function of this separate class of putative SMT but it is intriguing to speculate that their occurrence is related to the *E* stereochemistry of fucosterol that is found in diatom and red algae in contrast with the *Z* configuration of isofucosterol that is common in plants.

In plants and microalgae, C5-sterol desaturase (DWARF7), 7-dehydrocholesterol reductase (DWARF5) and 24-sterol reductase (DWARF1) control the latest steps in the pathway for isomerization of the double bond in the B-ring and reduction of C24(C28)-double bond in the alkyl chain. Transcripts of *S. marinoi* and *C. cryptica* showed sequences homologous to DWARF7 and DWARF5 that grouped with putative proteins of other diatoms (similarities above 40%) and a query coverage ranged from 50% to 86% to proteins of *A. thaliana*. In particular, At-DWARF7 and At-DWARF5 are homologous to the putative proteins Sm-TR28093 and Sm-TR2636 of *S. marinoi*, as well as to Cc-TR48724 and Cc-TR24068 of *C. cryptica*. In agreement with these results, un-rooted phylogenetic trees constructed using DWARF7/C5SR/ERG3 and DWARF5/7DHCR from plants, animals, fungi and yeasts showed clusterization of diatom sequences in the plant branch (Supplementary Figs. S7 and S8).

**Figure 5:**
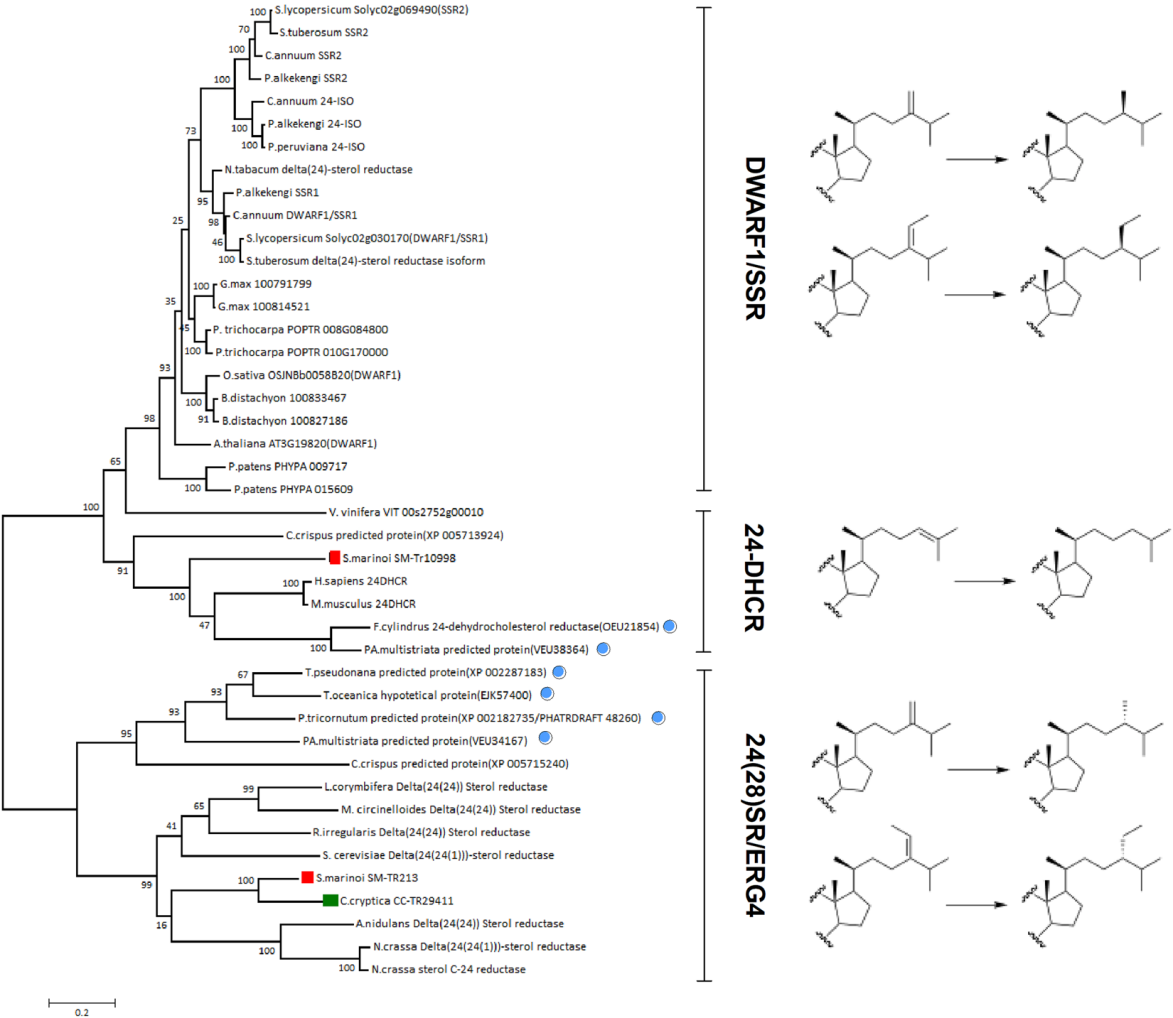
Un-rooted phylogenetic tree of 24-sterol reductases in diatoms, plants, algae, fungi and animals. Reactions catalyzed by each sub-family are shown next to the enzymatic groups. Alignment used maximum likelihood method. The bootstrapping test (replicate = 100) is reported on each node. Red square = *S. marinoi*; Green square = *C. cryptica*; Light blue spot = other diatom. DAWRF1 = plant 24-sterol reductase; SSR = plant sterol side chain reductase; 24-DHCR = animal 24-dehydrocholesterol reductase; 24(28)SR/ERG4 = fungal Delta(24(24)1))-sterol reductase.

Transcripts did not identify significant match with plant DWARF1 but indicated the presence of two putative sequences (Sm-TR213 and Cc-TR29411) with high similarity with Delta-(24)-sterol reductase (ERG4) from *Saccharomyces cerevisiae* and other fungi and yeasts (Fig. 5). Comparison versus different diatoms (*T. oceanica, T. pseudonana, P. tricornutum* and *F. cylindrus)* identified orthologous sequences of Sm-TR213 and Cc-TR29411, thus suggesting the wide occurrence and function of these genes in the lineage. Interestingly, the divergence of the diatom transcripts from the plant pathway for the reduction of the alkenyl group at C-24 is consistent with the β-stereochemistry of dihydrobrassicasterol and clionasterol in comparison to the α-orientation of campesterol and β-sitosterol in plants (Fig. 5). We have not been able to establish an unambiguous identification Δ_22_ sterols (trace components) in our study even if the molecular analysis of *C. cryptica* showed a transcript related to fungal 22-sterol reductase (ERG5) (Table 2).

### Biosynthesis of cholesterol and desmosterol

In plants, cholesterol and related sterols are little common. Only recently, a biosynthetic pathway of cholesterol has been proposed on the basis of functional assays including gene silencing and reactivity of recombinant tomato proteins^7^. According to this study, plant cholesterogenesis follows the Kandutsch-Russell pathways based on enzymes derived from the phytosterol biosynthesis after methylation of cycloartenol by sterol side chain reductase (SSR2)^56^. In addition to phytosterols, *S. marinoi* produces desmosterol and cholesterol, while these products are absent in *C. cryptica*. Desmosterol is directly converted to cholesterol by 24-DHCR in animals but it has been seldom reported in plants. For these reasons, *S. marinoi* and *C. cryptica* can serve as useful models to study the cholesterol biosynthesis in diatoms and, in analogy to plants, to investigate whether enzymes of the phytosterol pathway are possibly utilized for cholesterogenesis in this microalgal lineage.

Differential analysis of the two diatoms identified a sequence of a putative 24-DHCR (Sm-TR10998) that was present only in *S. marinoi*. Interestingly, Sm-TR10998 showed high similarity with the human 24-DHCR (Query coverage 94.7%; Positivies 67.4%) and, at less extent, with SSR2 of tomato (*Solanum lycopersicum*, Query coverage 92%; Positivies 53%) (Supplementary Fig. S7). No similar sequences were found in *C. cryptica*. As showed in the un-rooted phylogenetic tree of Fig. 5, Sm-TR10998 clustered in the 24-DHCR branch together with homologous sequences of the diatoms *F. cylindrus* (NCBI accession number: OEU21854) and *Pseudo-nitzschia multistriata (*NCBI accession number: VEU38364*)* that are both able to biosynthesize cholesterol^32^. In agreement with the evolutionary origin of diatoms, an ortholog of Sm-TR10998 was found in the red alga *C. crispus (*NCBI accession number: XP_005713924) that biosynthesizes sterol mixtures containing up to 94% of cholesterol^57^.

## Discussion

**Figure 6:**
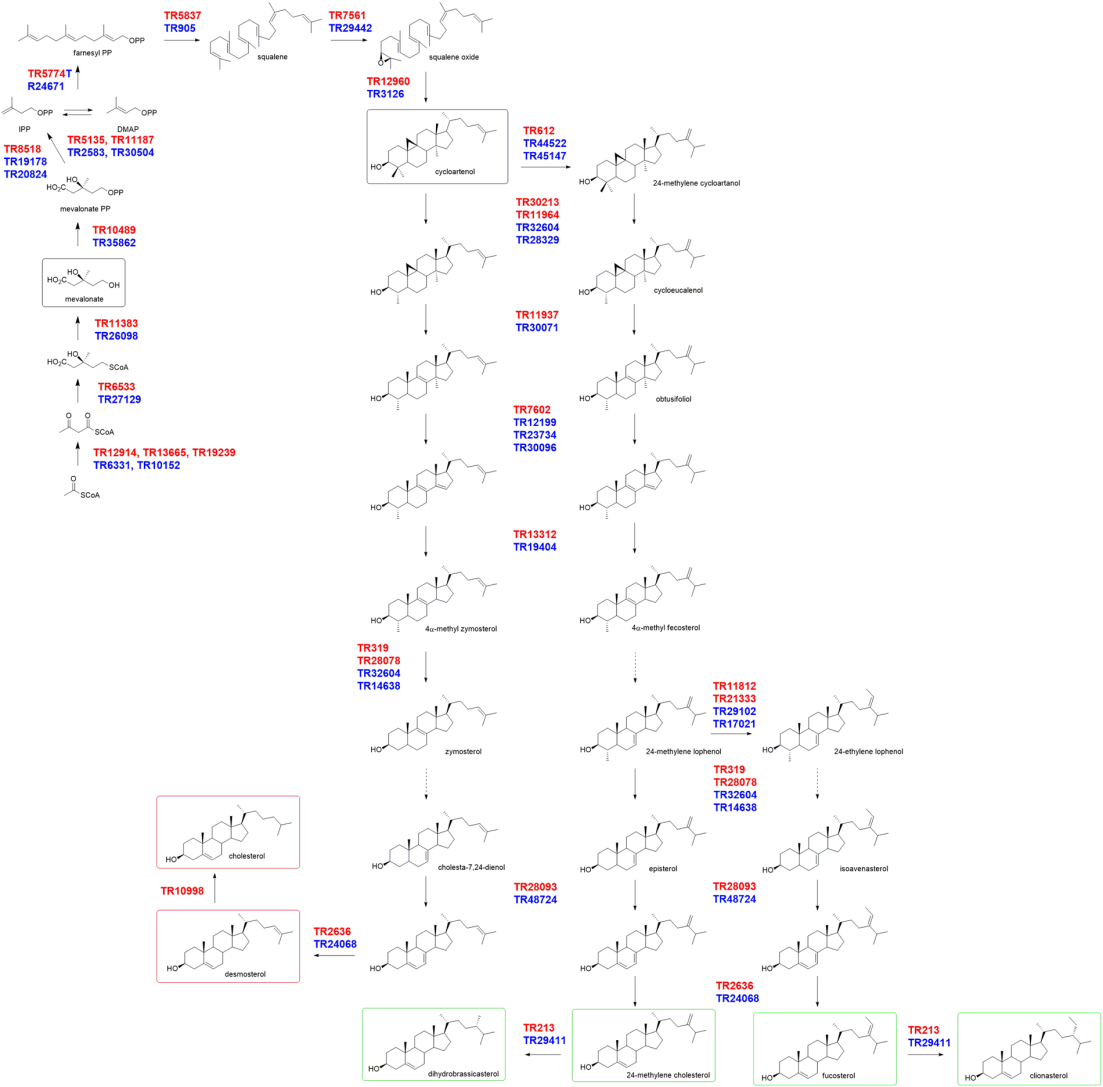
Proposed biosynthetic pathway of sterols in diatoms. Transcript sequences (Red = *S. marinoi*; Blue = *C. cryptica*) are in agreement with Table 1 and deposited under accession code SRP108217 and PRJNA561910. Red square = mammal sterols; Green square = phytosterols.

*C. cryptica* and *S. marinoi* are cosmopolitan diatom species that contribute significantly to phytoplankton blooms in temperate oceans. GC-MS profiling revealed a common mixture of sterols that is dominated by phytosterols with presence of cholesterol and desmosterol only in the latter species. These data are consistent with the literature that indicates major presence of C_27_ sterols in radial centrics like *S. marinoi*^32^. As depicted in the biosynthetic proposal of Fig. 6, the route to the sterol skeleton of 24-methylene cholesterol, dihydrobrassicasterol, fucosterol and clionasterol from IPP and DMAPP can have high similarity with plants. 24-Methylene cholesterol is the most common sterol of diatoms^32^ and a key intermediate of the biosynthesis of phytosterols. Despite this, levels of 24-methylene cholesterol in plants are usually low while this metabolite together with fucosterol and clionasterol (also known as γ-sitosterol) has been often reported in algae and lower invertebrates^6,9^.

In the literature, there are contrasting data about the biosynthesis of the isoprene units in diatoms. In fact, while *Rhizosolenia setigera*, *P. tricornutum*, and *Nitzschia ovalis* are reported to use mevalonate (MVA), *Haslea ostrearia* shows evidence for methylerythritol (MEP) as precursor of sterols^58,59^. By feeding studies with labelled glucose, we proved origin of the sterol skeleton only by the MVA pathway in *C. cryptica* thus corroborating the results on desmosterol of *R. setigera* by incorporation of 1-^13^C acetate^58^. The results of the labelling experiments found a match in the transcriptome sequences which showed a complete and expressed MVA pathway in *C. cryptica* (Fig. 2). Significantly, orthologs of these genes were well represented in the transcriptome of *S. marinoi*, as well as were found in the genome of *T. oceanica, T. pseudonana, P. tricornutum and F. cylindrus* (Supplementary Table S2).

After conversion of farnesyl pyrophosphate to squalene, the key step of the process is cyclization to cycloartenol by cycloartenol synthase (CAS) *via* squalene oxide. *C. cryptica* and *S. marinoi* showed a single copy of a plant-like CAS and the presence of a terbinafine-insensitive SQE, named AltSQE, that has been recently proposed to catalyze synthesis of squalene oxide in *S. marinoi* and other diatoms^50^. The wide occurrence of 24-methylene cholesterol in diatoms^32^ is consistent with the suggestion that, like in plants, methylation of the sterol side chain occurs very early in the biosynthetic process. In agreement with this view, the transcriptome analyses indicated the presence of different orthologs of plant SMT1, which are presumably responsible for C-24 methylation of cycloartenol to oryzanol^33,60^. On the other hand, our results showed major differences between plants and diatoms for SMT2 that presides methylation of 24-methylenelophenol to yield C_28_ phytosterols. It is noteworthy that this divergence corresponds to the difference in the stereochemistry of fucosterol and isofucosterol (Fig. 4) that are the end products of these enzymes in diatoms and plants respectively. SMTs embrace a class of proteins that have inherited a high degree of sequence similarity from a common ancestor. Recently, Nes and coworkers have discussed the differences of SMTs in relation to substrate- and phylum-specificity in green algae^61^ Our comparative analysis suggests that diatoms and the red alga *C. crispus* contain SMT sequences that may have evolved independently from plants, green algae and fungi. It is possible that diatoms have acquired these genes by the red alga that was engulfed during the secondary endosymbiotic event that is common to all stramenophiles, as well as we cannot exclude that these “red SMTs” can accept substrates other than 24-methylenelophenol. Further studies are necessary to prove the catalytic function of these proteins and the substrate specificity. However, the absence of scrambling of methyl groups in fucosterol and clionasterol indicates a high affinity of these enzymes for the substrates.

The high and specific ^13^C-incorporation of phytosterols of *C. cryptica*, as well as the absence of isotopic scrambling, features the operation of a unique and straightforward route similar to brassinolide pathway. In support to this view, sequence alignment of the putative enzymes of *S. marinoi* and *C. cryptica* revealed high homology with the corresponding proteins of plants. We found only major divergences of the transcript sequences (Sm-TR213 and Cc-TR29411) related to the reduction steps leading from 24-methylene cholesterol and fucosterol to dihydrobrassicasterol and clionasterol, respectively. These latter compounds are common algal metabolites and, despite the structural similarities with campesterol and β-sitosterol of plants, are produced by stereochemical reduction that envisages a mechanism sharply different from that described for phytosterols^6^. Accordingly, Sm-TR213 and Cc-TR29411 show high homology with fungal ERG4 that catalyzes hydrogen attack on 24-*si* face of the Δ24(28) double bond, which is consistent with the β-stereochemistry of the methyl and ethyl groups of dihydrobrassicasterol and clionasterol in *S. marinoi* and *C. cryptica* (Fig. 5). On the basis of the phylogenetic results, we suggest that reduction of 24-methylene cholesterol and fucosterol can be carried out by a single fungal-like Δ24(28)-sterol reductase. A similar hypothesis has been also put forward for the reduction of 24-methylene cholesterol and isofucosterol by a single 24-sterol reductase in plants^6^. Differently from the report on *P. tricornutum*^33^, *S. marinoi* and *C. cryptica* do not synthesise Δ_22_ sterols (e.g. diatomsterol). It is possible that the Δ_22_ unsaturation can be introduced in the late steps of the pathway by a 22-sterol desaturase similar to ERG5 that was detected in the transcripts of *C. cryptica*. However, the absence of typical fungal sterols (e.g. ergosterol) together with the lack of other molecular proofs argue against a fungus-like process for the sterol biosynthesis in these diatoms.

Our data clearly indicated no lanosterol synthase genes in *S. marinoi* and *C. cryptica* thus highlighting origin of cholesterol and desmosterol from cycloartenol. This process has been recently proved in tomato that can synthesize C_27_, C_28_ and C_29_ sterols by a promiscuous pathway^7^. Interestingly, despite the phylogenetic distances between tomato and diatoms, a certain degree of similarities can be identified in the sequences of key enzymes of sterol biosynthesis (Supporting Material). In tomato the divergence between cholesterol and phytosterols stems from the conversion of cycloartenol to cycloartanol by sterol side chain reductase enzyme (SSR2)^56^. However, since this reaction occurs very early in the sequence of enzymatic steps leading to cholesterol, the pathway is not compatible with the synthesis of desmosterol. On the other hand, desmosterol can accumulate as terminal product in diatoms and red algae (e.g. *Porphyridium purpureum*). Desmosterol is mostly known as the immediate precursor of cholesterol in the Block pathway in animals including humans, as well as it is a common intermediate in the conversion of C_28_ and C_29_ plant sterols to cholesterol by the tobacco hornworm and other insects^11,62^. Transcriptome of *S. marinoi* indicated a sequence (Sm-TR10998) with a predicted function related to 24-DHCR from *Homo sapiens*. The enzyme reduces desmosterol to cholesterol with the highest affinity for sterol skeleton lacking methyl groups at C-4, like desmosterol and 7,24-cholestandiol^63^. Differently from the proposal role of SSR2 in tomato, this specificity and the finding of desmosterol in *S. marinoi* suggest occurrence of 24-reduction in the late steps of the pathway (Fig. 6). Orthologs of Sm-TR10998 occur in all known genomes of diatoms producing cholesterol^32^, thus highlighting a general role of this enzyme in the lineage.

In conclusion, we suggest that the sterol pathway of *S. marinoi* and *C. cryptica* preserves the general architecture recently described in Solanaceae plants with cyclization of squalene oxide to cycloartenol rather than lanosterol that is characteristic of the fungal genealogy^7^. Major differences seem to be related to the putative enzymes that preside modification, namely methylation and reduction, of the alkyl side chain by enzymes encoded by genes probably derived from algae, fungi and animals. The acquisition of these genes explains well the composition and the stereochemistry of sterols in diatoms, as well as confirms the metabolic plasticity of these microalgae that are evolved through multiple events of symbiosis and horizontal gene transfer. Further studies are in progress to correlate the molecular data with biochemical tests.

## Methods

### General

HPLC analyses have been performed on a Jasco system (PU-2089 Plus-Quaternary gradient pump equipped with a Jasco MD-2018 Plus photodiode array detector). Cell pellet were lyophilized by a Savan Micro Modulyo freeze dryer (Thermo Scientific, Austin, TX, USA). GC-MS analysis was carried out by an Ion-Trap Polaris Q MS instrument (Thermo) coupled to a Focus gas chromatograph (Thermo). ^1^H and ^13^C NMR spectra were recorded on Bruker DRX 600 spectrometer equipped with an inverse TCI CryoProbe. All chemicals and analytical grade solvents were purchased from Sigma Aldrich. Molecular and bioinformatics analyses were performed by Genomix 4Life s.r.l. (Baronissi, Salerno, Italy).

### Microalgae cultures

Stock cultures of the diatom *Skeletonema marinoi* (CCMP 2092), *Skeletonema costatum, Pseudonitzchia arenysensis* (B758), *Thalassiosira weissflogii* (P09), *Cyclothella criptica* (CCMP 331) and *Phaeodactylum tricornutum* were purchased from Bigelow Laboratories. Strains were maintained in f/2 medium^64^ in a growth chamber at 20 ± 2 °C under 14:10 light:dark cycle with a photon flux density of 100 μmol quanta m^−2^ sec^−1^. Cultures were harvested by centrifugation at 3750 rpm for 10 minutes at 12 °C using a swing-out rotor. Pellets were frozen in liquid nitrogen and stored at −80 °C until analysis.

### Labelling studies

For biosynthetic experiments, *C. criptica* was incubated in 1 L-sterile culture flasks (TPP25–300 cm^2^) in prefiltered sterile (0.22 µm) f/2 medium supplemented with antibiotics according to published protocols^65,66^. Under these conditions, cells were grown heterotrophically on 2 g L^−1^ of glucose or 1-^13^C-glucose in the dark at 20 ± 2 °C under constant gentle agitation. Cultures were harvested and stored at −80 °C until analysis, as described above.

### Sterol extraction and purification

After lyophilization, microalgal pellets were extracted by the modified Folch method. The oily residues were dried, reconstituted in diethyl ether and methylated by diazomethane (0.4 mL of a saturated ether solution per 10 mg extract). After removing the organic solvent under N_2_, sterols were purified on silica gel column (100 mg silica gel/ mg fraction). Homogeneous fractions of sterol mixtures were achieved in petroleum ether/diethyl ether 85:15 (v/v). Product elution was monitored by SiO_2_-TLC on 0.2-mm aluminum-coated sheets (Merck, Germany) developed with petroleum ether/ethyl ether (1:1; v/v). Final purification of sterols was carried out by HPLC on a reverse phase column (C18-Luna, Phenomenex, 5 μm 100 A 250 × 10 mm) by methanol/acetonitrile/water/iPrOHl/acetone (33:33:4:5:25; v-v) according to our published protocols^67,68^. Elution was accomplished by a flow of 1 mL min^−1^ and monitored by UV absorbance at 210 nm.

### Sterol analysis

For GC-MS analysis, sterols were acetylated by Ac_2_O (100 µL/ 0.5 mg sample) in dry pyridine (500 µL/0.5 mg sample) under magnetic stirring overnight at room temperature. The excess of organic solvents was removed under N_2_ stream. GC-MS runs of acetylated sterols were carried out in EI mode (70 eV) by isocratic elution at 300 °C for 30 min, with a 5% diphenyl-polysiloxane capillary column (OV-5 column) (15 m x 0.32 mm ID, 0.10 µm) and helium as gas carrier. Samples dissolved in n-hexane were directly injected (2 μL) in split mode (1:10), with a blink window of 3 minutes, inlet temperature of 300 ° C, transfer line set at 310 ° C and ion source temperature of 300 ° C. Sterols were identified according to literature and for comparison with commercial standards^35–40,69–72^. For NMR experiments (^1^H, ^13^C, COSY, TOCSY, HSQC), natural and standard sterols were dissolved in 700 µL CDCl_3_ and transferred to 5-mm NMR tube. For biosynthetic studies, ^13^C NMR spectra were acquired with a relaxation delay of 6 seconds to reduce the effect of longitudinal relaxation.

### Transcriptome analysis and bioinformatics

RNA was extracted by Trizol method and RNA sequencing experiment was performed on three biological replicates for diatoms. Before use, RNA concentration in each sample was assayed with a ND-1000 spectrophotometer (NanoDrop) and quality was assessed with the Agilent 2100 Bioanalyzer with Agilent RNA 6000 nano kit (Agilent Technologies, Santa Clara, CA, USA). Indexed libraries were prepared from 4 μg/ea purified RNA with TruSeq Stranded mRNA Sample Prep Kit (Illumina) according to the manufacturer’s instructions. Libraries were quantified using the Agilent 2100 Bioanalyzer (Agilent Technologies) and pooled such that each index-tagged sample was present in equimolar amounts, with a final concentration of the pooled samples of 2 nM. The pooled samples were subject to cluster generation and sequencing using an Illumina HiSeq. 2500 System (Illumina)in a 2 × 100 paired-end format at a final concentration of 8 pmol. Transcriptome was assembled using Trinity^73^. The high-quality reads were selected as input to perform the transcriptome assembly and the resulting sequences were translated into proteins by using the longest complete ORF. The sequences of the assembled transcripts were translated into proteins with Transdecoder and the software Blast2GO was used to associate a function to identified transcripts. To compute abundance estimation, the high-quality reads were aligned to the Trinity transcriptome using bowtie2. Then, either RSEM is executed to estimate expression values based on the resulting alignments, generating the raw counts. In order to define the set of expressed genes, raw read counts were normalized using the TMM method (Trimmed mean). Phylogenetic analyses were refined by MEGA 6.0 software^74^ and alignments were obtained by the MUSCLE algorithm. Phylogenetic trees were built by the maximum likelihood method with the LG + G substitution model (for CAS, 7DHCR/DWF5, SMT and 24-DHCR/DWF1) and LG + G + I (for C5SD/DWF7). Test of phylogeny was performed by the bootstrap method with a number of bootstrap replication equal to 100.

## Supplementary information

Supplementary Information and Supporting Material.

## Data Availability

The raw sequencing data from this study have been submitted to the NCBI SRA database (http://www.ncbi.nlm.nih.gov). The RNA sequencing data of *S. marinoi* and *C. cryptica* have been deposited under accession code SRP108217 and PRJNA561910. The sterol related sequences of interest are reported in the Supplementary Material. All other supporting data from this study are available within the article and its Supplementary Information Files, or from the corresponding authors upon request.
