# Supplementary material for: Diatoms synthesize sterols by inclusion of animal and fungal genes in the plant pathway
Source: Sci Rep. 2020 Mar 6;10:4204. doi: 10.1038/s41598-020-60993-5 (PMC7060231; doi:10.1038/s41598-020-60993-5)
Supplement: Supplementary file 1 — Supplementary Information and Supporting Material. [file 41598_2020_60993_MOESM1_ESM.pdf]

SUPPLEMENTARY INFORMATION

Diatoms synthesize sterols by inclusion of animal and fungal genes in the plant pathway

Carmela Gallo[1], Simone Landi[1,2], Giuliana d'Ippolito[1], Genoveffa Nuzzo[1], Emiliano Manzo[1], Angela Sardo[1,3], Angelo Fontana[1]

[1] National Research Council of Italy, Institute of Biomolecular Chemistry, Bio-Organic Chemistry Unit, Via Campi Flegrei 34, 80078 Pozzuoli (Napoli), Italy.

[2] Univeristy of Naples "Federico II", Department of Biology, Cupa Nuova Cintia 21 , 80126 Napoli, Italy

[3] Stazione Zoologica "A. Dohrn", Villa Comunale, 80121 Napoli, Italy

Supplementary Table 1: Sterol composition of centric and pennate diatoms based on the 5-cholesten skeleton

**Diatom species**	**Phytosterols**					**Animal sterols**	
	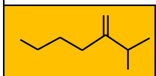	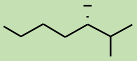	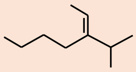	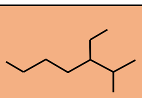	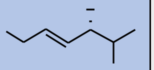	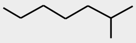	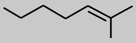
Cyclotella cryptica[a]							
Skeletonema marinoi[a]							
Skeletonema costatum[b]							
Thalassiosira weissflogii[a]							
Pseudonitzschia arenysensis[a]							
Phaeodactylum tricornutum[a]							
Phaeodactylum tricornutum[b]							
Thalassiosira pseudonana[b]							
Cylindrotheca closterium[b]							
Fragilariopsis cylindrus[b]							

[a]Current study; [b]In agreement with Limnol. Oceanogr., 55(1), 2010, 91–105

Supplementary Table 2: List of main identified orthologs obtained using nodal sterol pathway translated sequences from *S. marinoi* and *C. cryptica* compared *vs T. oceanica, T. pseudonana, F. cylindrus, P. tricornutum, PN. multistriata and C. crispus* genomes. In addition, the sequence of the ERG2 from *Saccharomyces cerevisiae* was used as further query.

		**Main identified orthologs in sequenced genomes of marine diatoms**											
**Sterol biosynthesis key genes**		***T.oceanica***	**Identity**	***T.pseudonana***	**Identity**	***F.cylindrus***	**Identity**	***P.tricornutum***	**Identity**	***PN.multistriata***	**Identity**	***C.crispus***	**Identity**
HMG-CoA synthase	HMGS	EJK77540.1	84%	XP_002286567.1	87%	OEU16766.1	72%	XP_002185047.1	65%	VEU40541.1	65%	XP_005712048.1	37%
HMG-CoA reductase	HMGR	EJK68891.1	73%	XP_002289576.1	89%	OEU16221.1	78%	XP_002185302.1	74%	not determined		not determined	
Mevalonate kinase	MEVK	EJK44829.1	75%	XP_002287787.1	83%	OEU13577.1	69%	XP_002177012.1	26%	VEU38327.1	69%	XP_005711370.1	37%
Squalene synthase	SQS	EJK68341.1	77%	XP_002288355.1	88%	OEU14974.1	70%	XP_002180940.1	72%	VEU34553.1	65%	XP_005712480.1	36%
Cycloartenol synthase	CAS	EJK52279.1	64%	XP_002287432.1	85%	OEU13816.1	68%	XP_002185678.1	71%	VEU33723.1	57%	XP_005715573.1	45%
Cycloartenol-C-24-methyltransferase	SMT1	EJK49936.1	37%	XP_002287743.1	69%	OEU12190.1	50%	XP_002178531.1	42%	VEU39293.1	26%	XP_005710912.1	25%
Cycloeucalenol cycloisomerase	CPI	EJK52525.1	68%	XP_002291862.1	72%	OEU13221.1	52%	XP_002184217.1	59%	VEU40796.1	52%	XP_005714180.1	41%
Sterol 14 demethylase	CYP	EJK78043.1	81%	XP_002289823.1	91%	OEU16403.1	59%	XP_002185538.1	65%	VEU38469.1	61%	XP_005711653.1	46%
Delta 14 sterol reductase	CYP	EJK57401.1	34%	XP_002287678.1	74%	OEU08604.1	56%	XP_002183899.1	35%	VEU34167.1	51%	XP_005715240.1	33%
C8 sterol isomerase (ScERG2)	ERG2											XP_005718833.1	47%
C-5 sterol desaturase	DWF7	EJK66009.1	38%	XP_002295550.1	80%	OEU21245.1	51%	XP_002179834.1	55%	VEU39255.1	65%	XP_005719132.1	30%
7 dehydrocholesterol reductase	DWF5	EJK73338.1	76%	XP_002289717.1	79%	OEU22434.1	70%	XP_002184305.1	38%	VEU39255.1	63%	XP_005719145.1	34%
24 dehydrocholesterol reductase (Sm-TR10988)	DHCR24					OEU21854.1	43%			VEU38364.1	42%	XP_005713924.1	35%
Delta(24(24(1)))-sterol reductase	24SR	EJK57400.1	30%	XP_002287183.1	31%	OEU08604.1	35%	XP_002182735.1	32%	VEU34167.1	31%	XP_005715240.1	29%

Supplementary Table 3: Comparison analysis of plants, fungal, yeast and human enzymes involved key reaction of sterol synthesis *vs* the *S. marinoi* and *C. cryptica* sequenced transcriptomes. Resulted hits showing alignment query coverage ≤ 39% were considered not significant and dark grey highlighted.

			Best BLAST hit			
**Organisms**	**Descriptions**	**Genes**	***S. marinoi***	**Statistics**	***C. cryptica***	**Statistics**
Arabidopsis thaliana	*Squalene epoxidase*	SQE1	No hit		No hit	
Homo sapiens	*Squalene epoxidase*	SQE	No hit		No hit	
Pheodactylum tricornutm	*Alternative squalene epoxidase*	AltSQE	TR7561	QC = 58% I = 25% P= 42%	TR29442	QC = 50% I = 26% P= 43%
Arabidopsis thaliana	*Cycloarthenol synthase*	CAS1	TR12960	QC = 86% I = 47% P= 64%	TR3126	QC = 86% I = 48% P= 64%
Neurospora crassa	*Lanosterol synthase*	LSS		QC = 92% I = 38% P= 54%		QC = 86% I = 39% P= 55%
Homo sapiens	*Lanosterol synthase*	LSS		QC = 89% I = 44% P= 60%		QC = 90% I = 45% P= 60%
Aspergillus nidulans	*Lanosterol synthase*	LSS		QC = 87% I = 32% P= 49%		QC = 87% I = 32% P= 48%
Arabidopsis thaliana	*Sterol 14 demethylase*	CYP10A1	TR7602	QC = 76% I = 25% P= 43%	TR12199	QC = 77% I = 25% P= 41%
Arabidopsis thaliana	*Sterol 14 demethylase*	CYP10A2		QC = 41% I = 29% P= 48%		QC = 42% I = 30% P= 31%
Arabidopsis thaliana	*C8-sterol isomerase*	AT1G05440	TR11754	QC = 15% I = 23% P= 51%	No hit	
Arabidopsis thaliana	*C8-sterol isomerase*	HYD1	TR27203	QC = 24% I = 23% P= 55%	TR38445	QC = 23% I = 41% P= 50%
Saccharomyces cerevisiae	*C8-sterol isomerase*	ERG2	TR4901	QC = 22% I = 29% P= 57%	TR41732	QC = 18% I = 32% P= 51%
Aspergillus nidulans	*C8-sterol isomerase*	ERG2	TR2164	QC = 12% I = 42% P= 70%	TR9781	QC = 19% I = 36% P= 54%
Neurospora crassa	*C8-sterol isomerase*	ERG2	TR4901	QC = 22% I = 29% P= 57%	TR17006	QC = 21% I = 27% P= 45%
Arabidopsis thaliana	*Sterol methyltransferase 1*	SMT1	TR612	QC = 89% I = 46% P= 65%	TR44522	QC = 98% I = 43% P= 53%
Chlamydomonas reinhardtii	*Sterol methyltransferase*	SMT1-2	TR612	QC = 84% I = 38% P= 53%	TR45147	QC = 84% I = 40% P= 53%
Arabidopsis thaliana	*Sterol methyl-oxidase*	SMO1-1	TR11964	QC = 62% I = 27% P= 43%	TR32604	QC = 60% I = 31% P= 45%
Arabidopsis thaliana	*Sterol methyl-oxidase*	SMO1-2	TR30213	QC = 81% I = 26% P= 45%		QC = 80% I = 23% P= 42%
Arabidopsis thaliana	*Sterol methyl-oxidase*	SMO1-3	TR11964	QC = 70% I = 27% P= 45%		QC = 56% I = 30% P= 45%
Arabidopsis thaliana	*Sterol methyl-oxidase*	SMO2-1	TR319	QC = 84% I = 23% P= 40%		QC = 58% I = 25% P= 52%
Arabidopsis thaliana	*Sterol methyl-oxidase*	SMO2-2	TR28078	QC = 78% I = 28% P= 46%		QC = 59% I = 33% P= 52%
Homo sapiens	*C4 methyl sterol oxidase*	SMO	TR11964	QC = 86% I = 25% P= 43%		QC = 75% I = 24% P= 45%
Saccharomyces cerevisiae	*Sterol-4-alpha-carboxylate 3-dehydrogenase*	ERG26	TR28078	QC = 98% I = 25% P= 42%	TR14638	QC = 98% I = 29% P= 48%

Homo sapiens	*3-beta-hydroxysteroid-Delta(8),Delta(7)-isomerase*	EBP	No hit		No hit	
Arabidopsis thaliana	*C5-sterol desaturase*	DWF7	TR28093	QC = 58% I = 23% P= 42%	TR48724	QC = 51% I = 25% P= 43%
Homo sapiens	*C5-sterol desaturase*	SC5D	TR28093	QC = 79% I = 25% P= 43%	TR48724	QC = 48% I = 32% P= 43%
Arabidopsis thaliana	*7-dehydrocholesterol reductase*	DWF5	TR2636	QC = 86% I = 40% P= 55%	TR24068	QC = 86% I = 41% P= 56%
Homo sapiens	*24-dehydrocholesterol reductase*	DHCR24	TR10998	QC = 95% I = 50% P= 67%	TR28124	QC = 24% I = 25% P= 44%
Arabidopsis thaliana	*Delta 24 sterol reductase*	DWF1		QC = 92% I = 37% P= 54%		QC = 11% I = 33% P= 52%
Solanum lycopersicum	*Sterol side chain reductase*	SSR2		QC = 92% I = 39% P= 53%		QC = 25% I = 25% P= 42%
Physalis alkekengi	*Delta 24 sterol reductase*	24ISO		QC = 91% I = 39% P= 53%		QC = 23% I = 25% P= 45%
Saccharomyces cerevisiae	*Delta 24(24) sterol reductase*	ERG4	TR213	QC = 94% I = 43% P= 59%	TR29411	QC = 93% I = 43% P= 58%
Saccharomyces cerevisiae	*Delta 22 sterol desaturase*	ERG5	TR7602	QC = 69% I = 22% P= 49%	TR3228	QC = 40% I = 28% P= 43%

Supplementary Figure 1:NMR (600 MHz, $CDCl_3$) identification of clionasterol from *S. marinoi* and *C. cryptica*. (**A**) Comparison of ^{1}H NMR upfield-shifted signals of standard β sitosterol and the natural 24 ethyl sterol isolated from the diatoms; (**B**) ^{1}H-NMR data of clionasterol and β-sitosterol, and comparison with the literature assignment (Nes *et al.* Lipids (1976) 11: 118).

A)

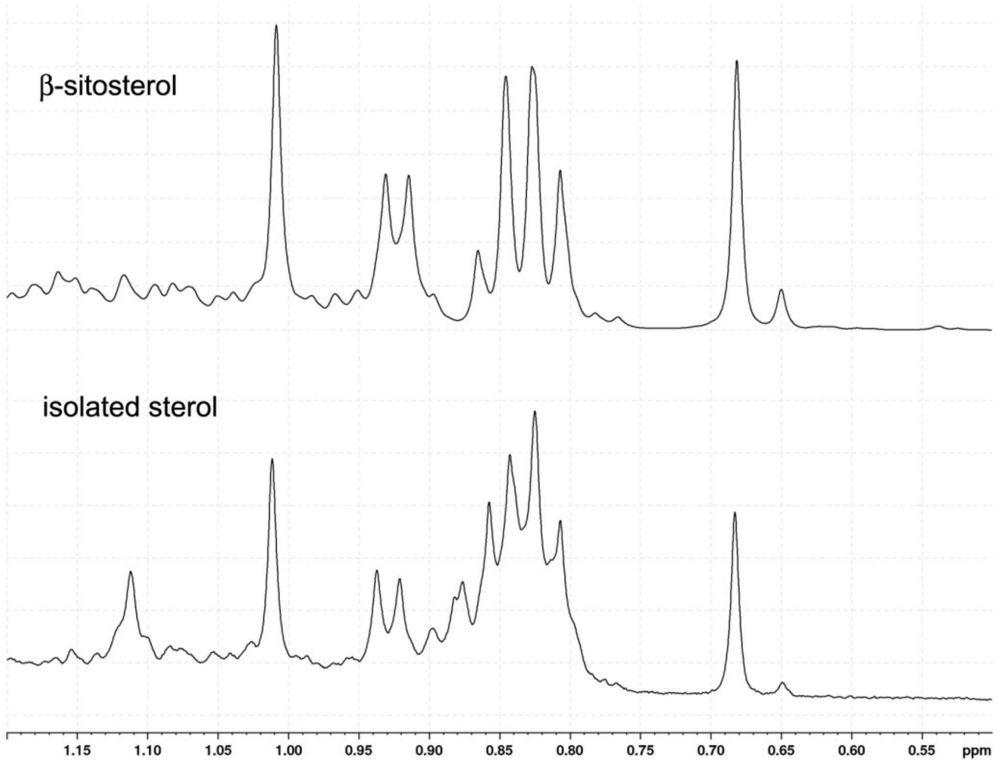

B)

	Experimental data		Literature data	
Positions	Clionasterol[a]	β-Sitosterol[b]	Clionasterol	β-Sitosterol
18	0.6800	0.6826	0.6818	0.6818
19	1.0090	1.0090	1.0090	1.0090
21	0.9247	0.9243	0.9272	0.9227
26	0.8308	0.8371	0.8318	0.8364
27	0.8106	0.8163	0.8091	0.8136
29	0.8578	0.8462	0.8545	0.8454

[a] sample isolated from *C. cryptica*; [b] standard.

Supplementary Figure 2: Incorporation of 1-^{13}C-glucose into dihydrobrassicasterol of *C. cryptica.* Enriched carbons are shown by a red spot on the structure. (**A**) Full and (**B**-**D**) four enlarged regions of the ^{13}C-NMR spectra ($CDCl_3$, 125 MHz) of labeled (top trace) and unlabeled (down trace) dihydrobrassicasterol. Intensity of ^{13}C signals of labeled and unlabeled spectra are normalized on C14 (56.79 ppm).

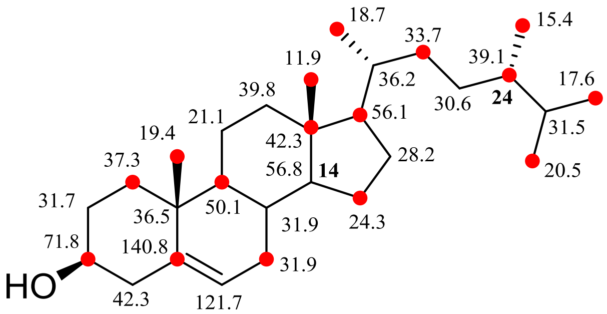

A) Full spectral width (155-0 ppm).

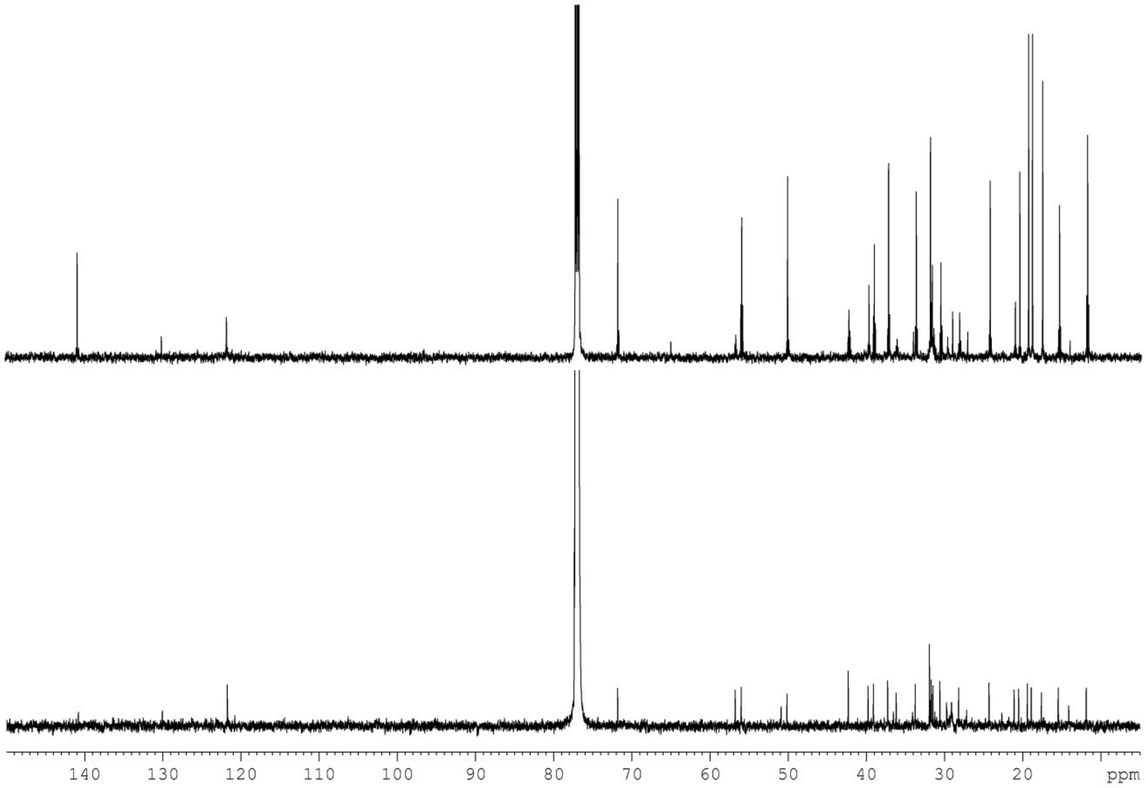

B) Region between 35 and 10 ppm.

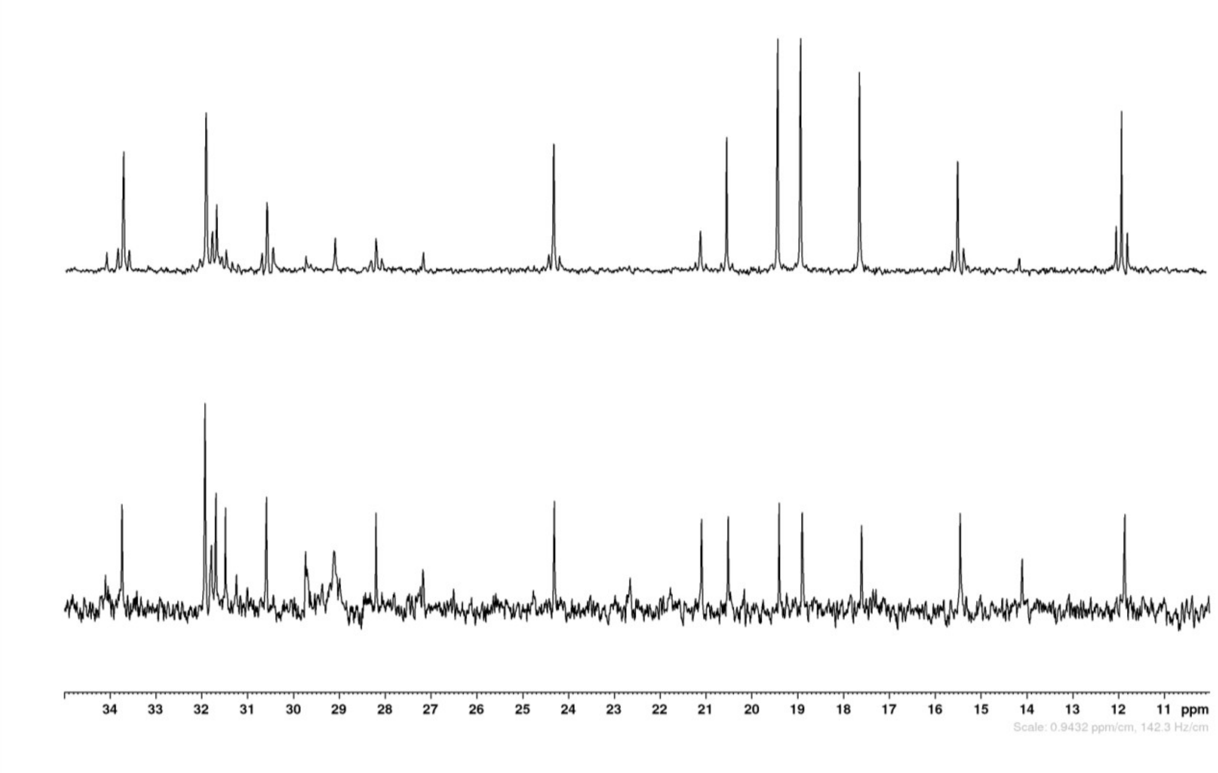

C) Region between 60 and 35 ppm.

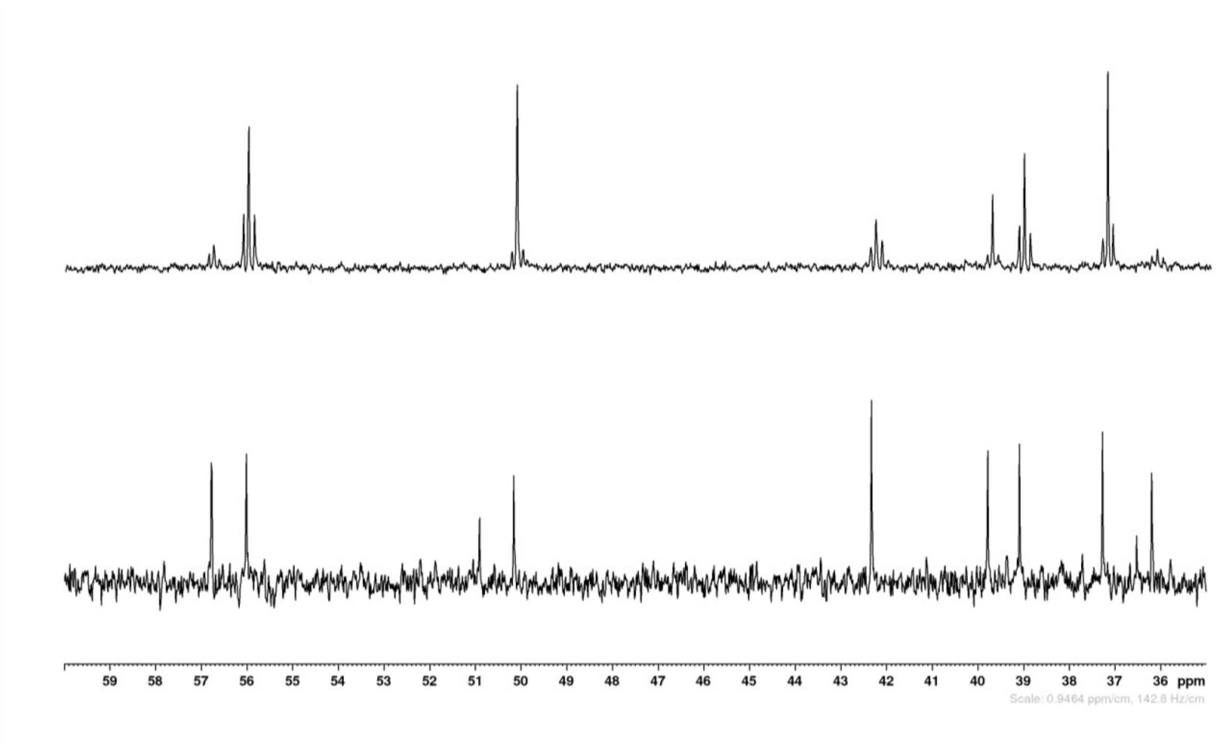

D) Region between 85 and 60 ppm.

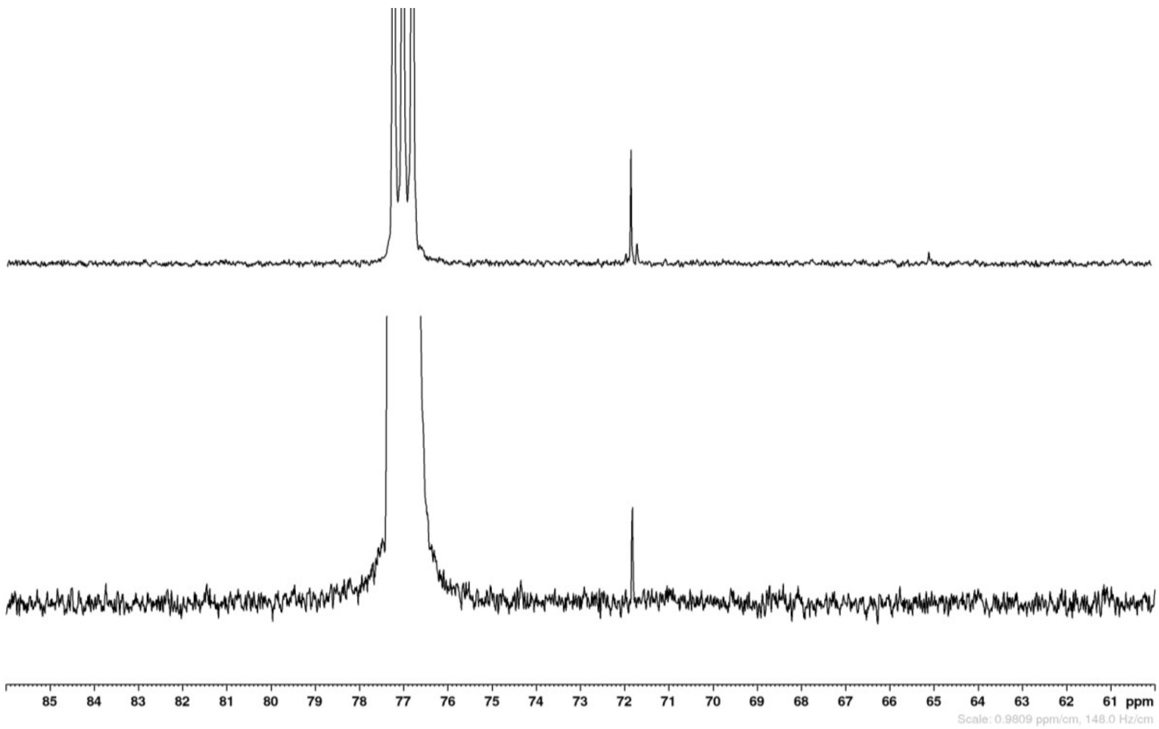

E) Region between 144 and 118 ppm

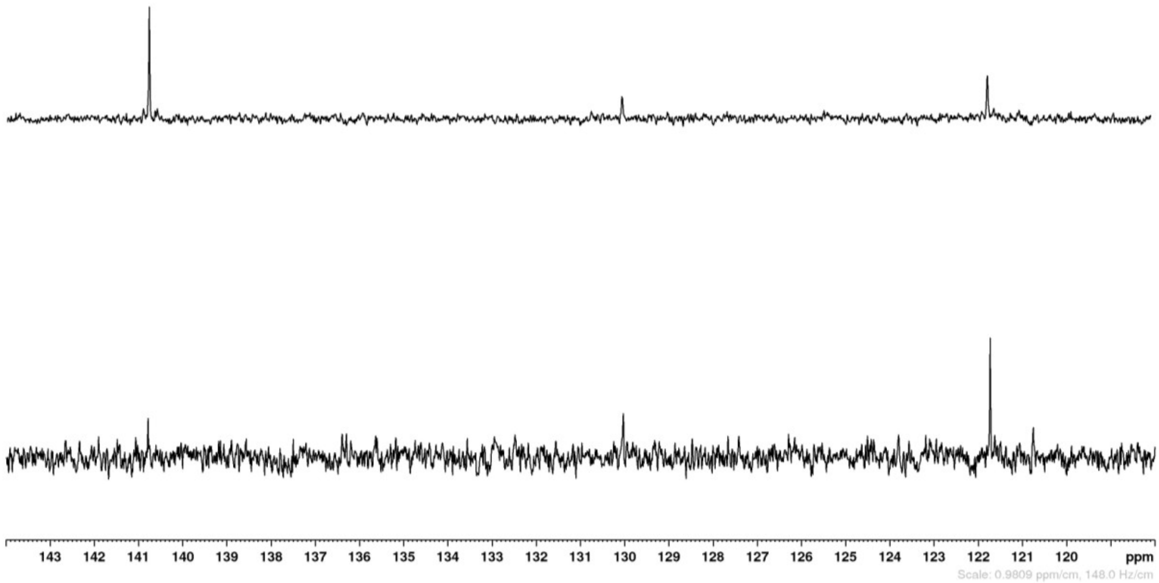

Supplementary Figure 3: Incorporation of 1-^{13}C glucose into fucosterol of *C. cryptica.* Enriched carbons are shown by a red spot on the structure. (**A**) Full and (**B**-**D**) enlarged regions of the ^{13}C-NMR spectra ($CDCl_3$, 125 MHz) of labeled (top trace) and unlabeled (down trace) fucosterol. Intensity of ^{13}C signals of labeled and unlabeled spectra are normalized on C14 (56.79 ppm).

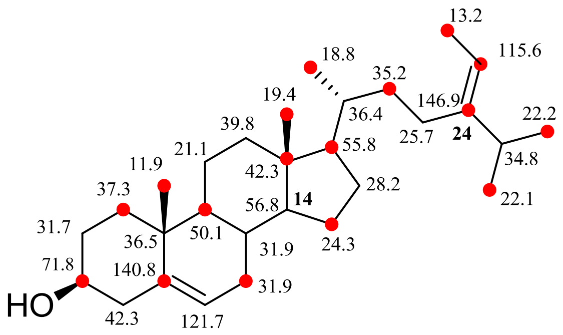

A) Full spectral width (160-0 ppm).

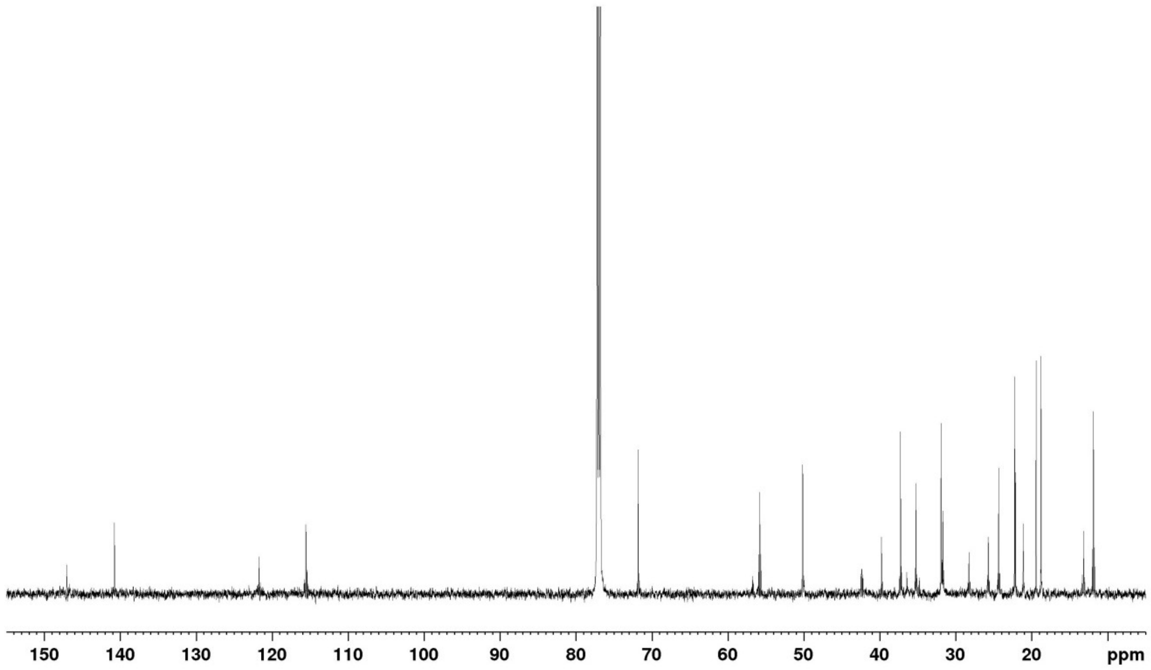

B) Region between 35 and 10 ppm.

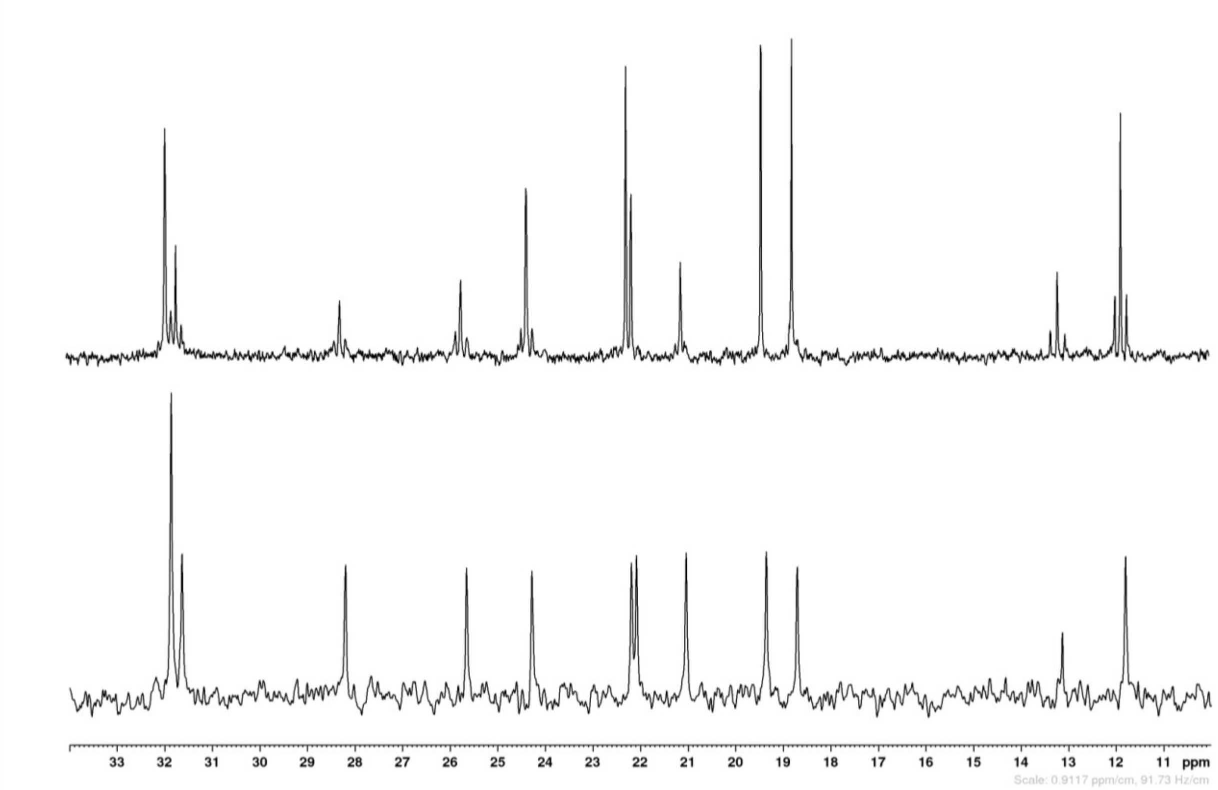

C) Region between 60 and 35 ppm.

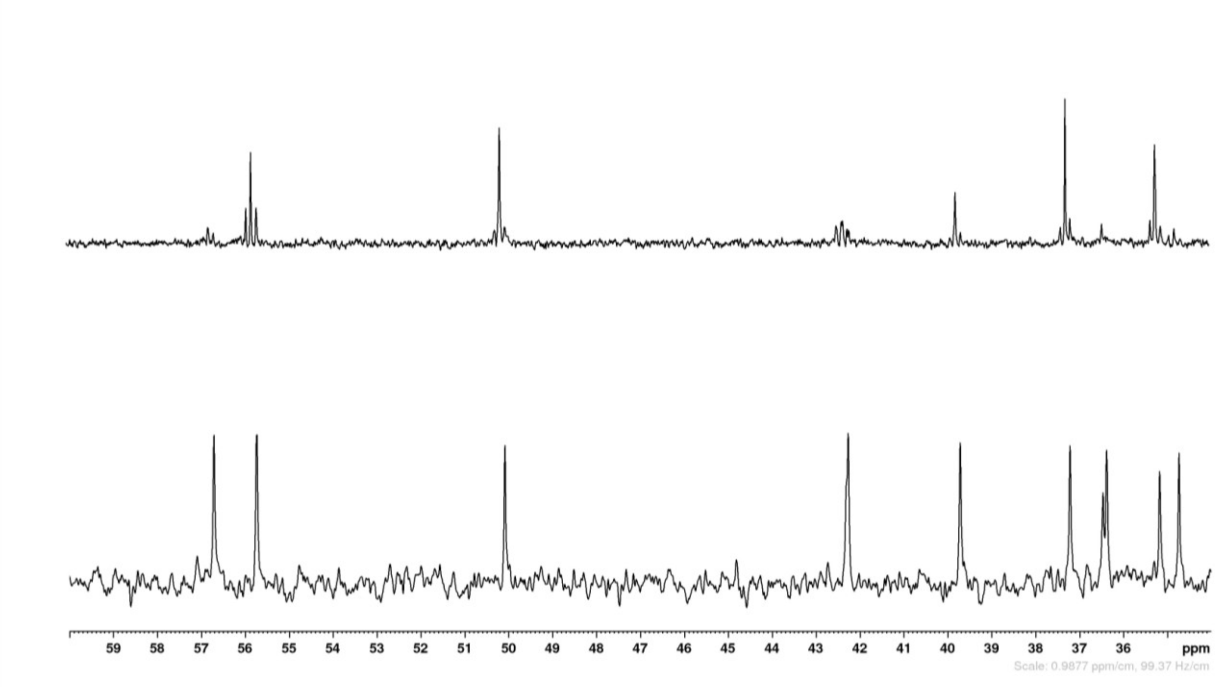

D) Region between 85 and 60 ppm.

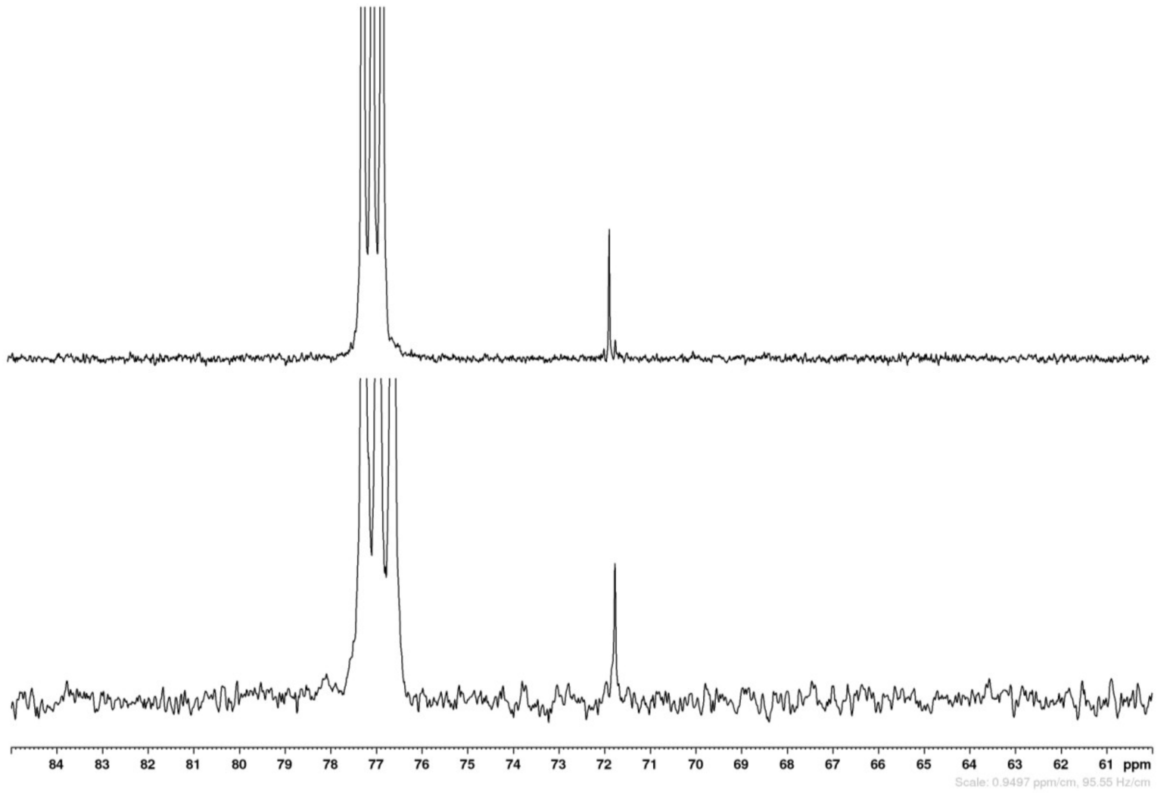

E) Region between 150 and 110 ppm.

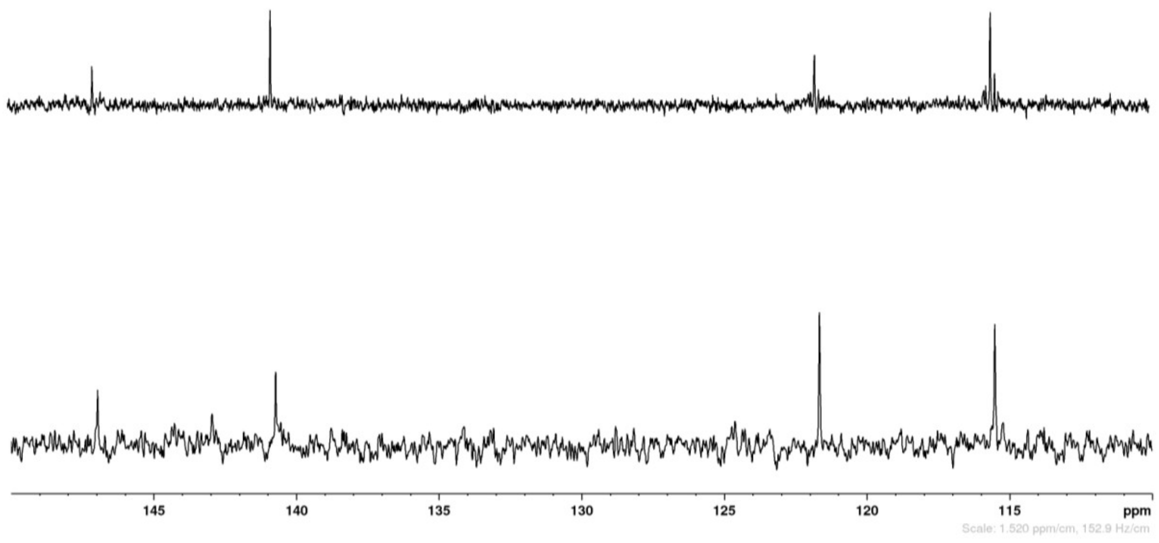

Supplementary Figure 4: Incorporation of 1-^{13}C glucose into clionasterol of *C. cryptica.* Enriched carbons are shown by a red spot on the structure. (**A**) Full and (**B**-**D**) enlarged regions of the ^{13}C-NMR spectra ($CDCl_3$, 125 MHz) of labeled clionasterol.

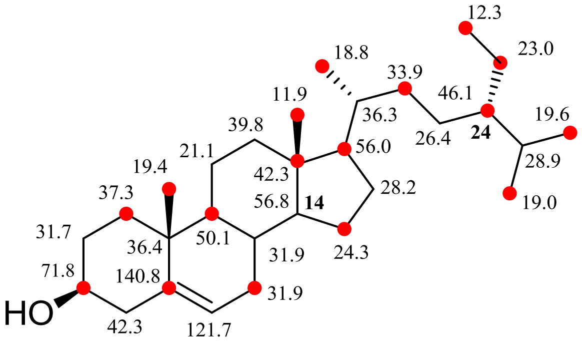

A) Full spectral width (160-0 ppm).

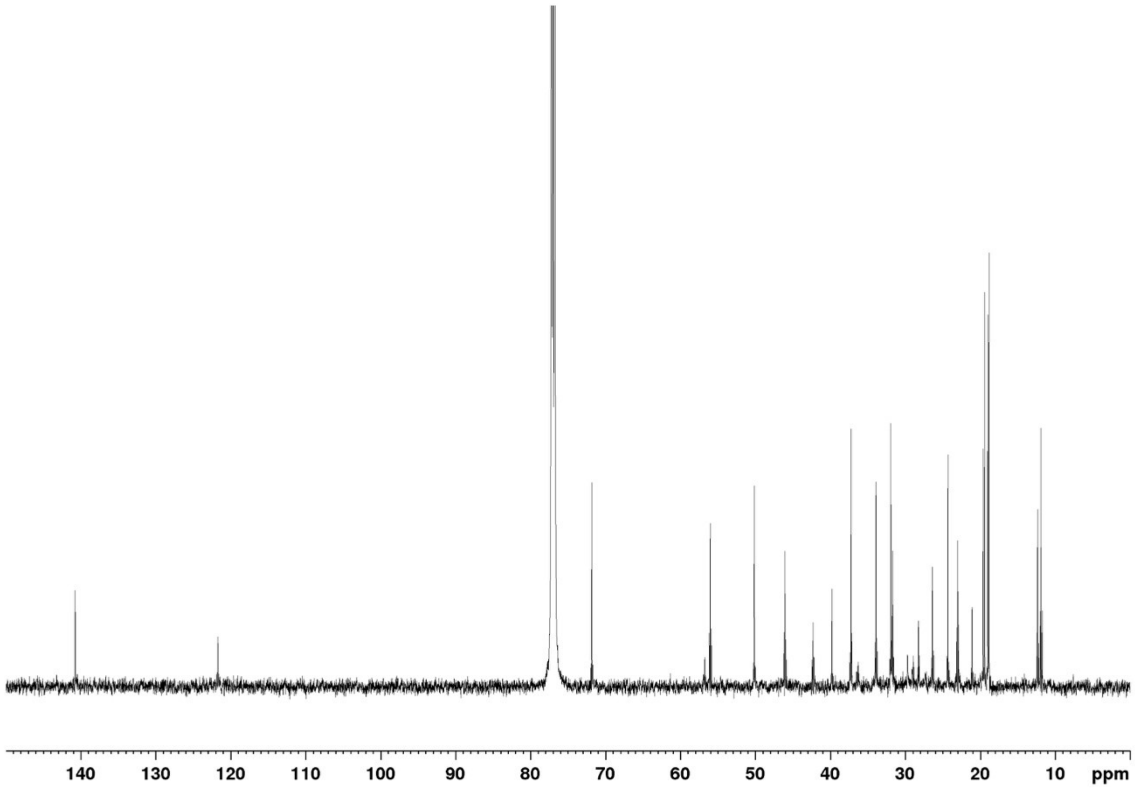

B) Region between 35 and 10 ppm.

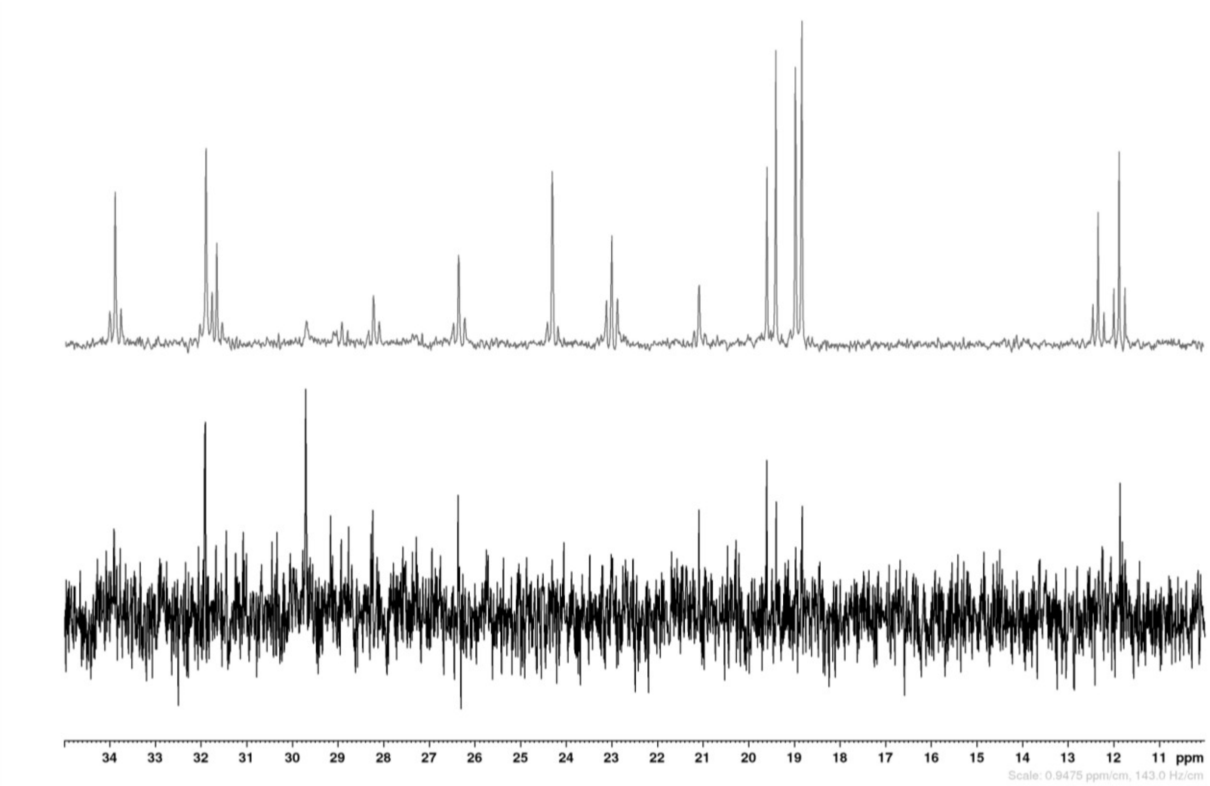

C) Region between 60 and 35 ppm.

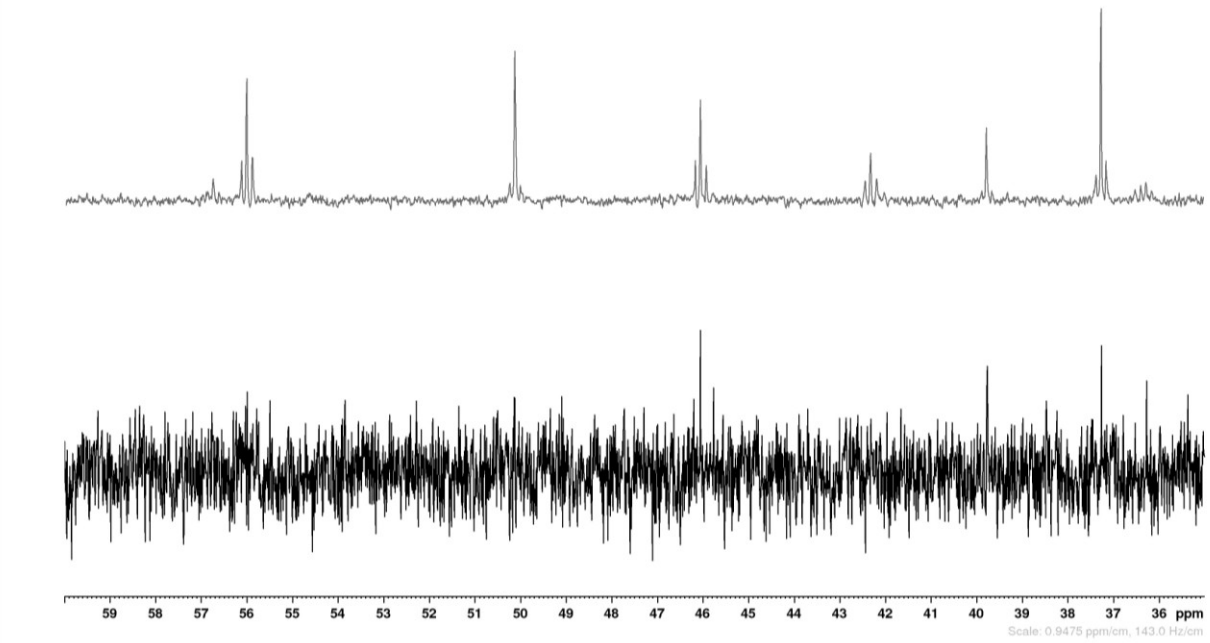

D) Region between 85 and 60 ppm.

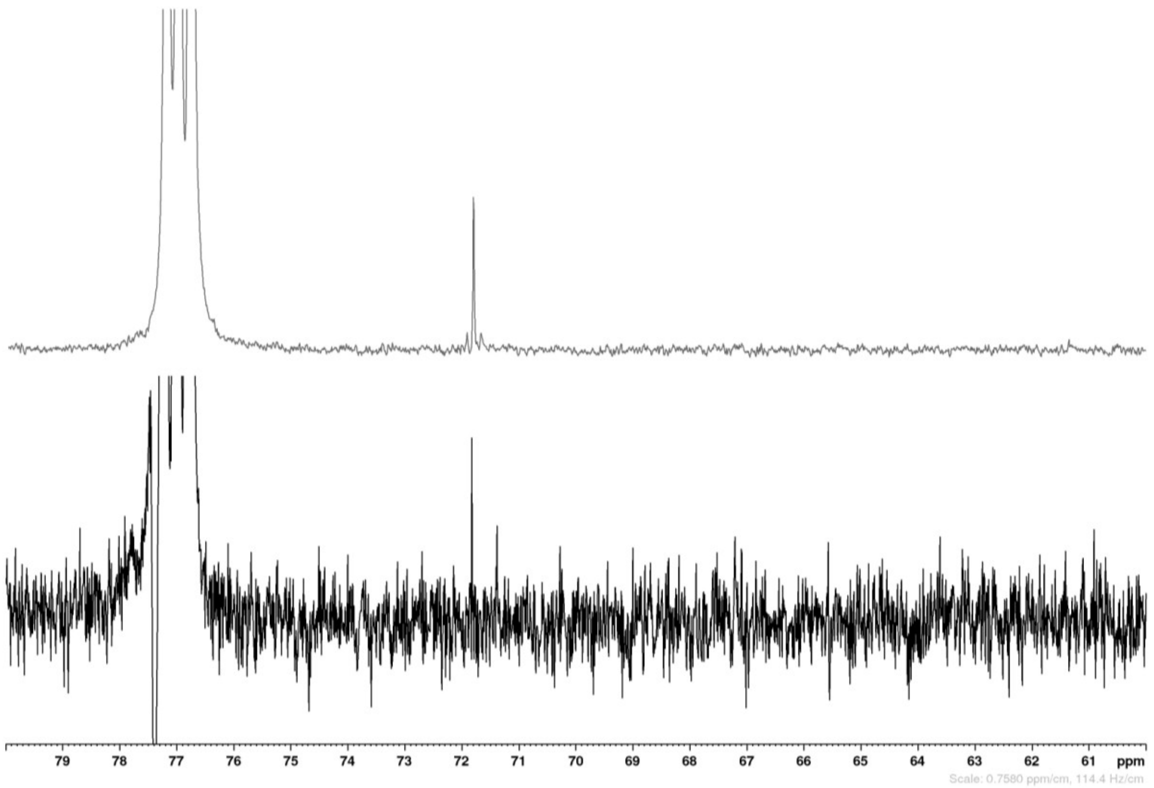

E) Region between 145 and 118 ppm.

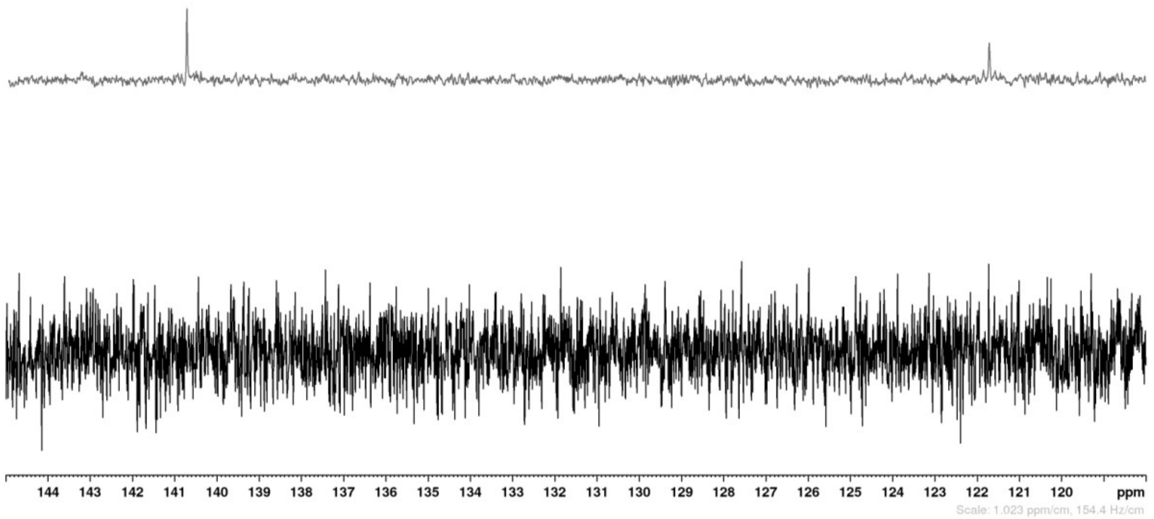

Supplementary Figure 5: Alignment of amino acid sequences of the catalytic region of the putative proteins of *S. marinoi* and *C. cryptica* with homologous regions from CAS and LSS in animals, yeast and fungi.

```
A.nidulans_LSS       342 DSNDLTNSTGSVSVDAFLNMIAFYC--KEGPDSKALRRIQTASLEYLWMGPRGMQVMSIH 399
H.sapiens_LSS        325 ADDRFTKSISIGPISKTINMLVRWY--VDGPASTAFQEHVSRIPDYLWMGLDGMKMQGTN 382
A.thaliana_CAS1      355 YEDENTRYICIGPVNKVLNMLCCWV---EDPNSEAFKLHLPRIHDFLWLAEDGMKMQGYN 411
A.thaliana_CAS2      264 YEDENTRYICIGPVNKVLNMLCCWV---EDPNSEAFKLHLPRIHDFLWLAEDGMKMQGYN 320
S.marinoi_CAS        776 AEDLQTNFIDIGPVNKALNMVSAFHSANNDINDPAVRSHMMRVPDYLWVAEDGMKMQGYN 835
C.criptica_CAS       299 AEDLQTNYIDIGPVNKALNMVAAYHAANNDINDPAVQSHMMRVPDYLWLAEDGMKMQGYN 358
N.crassa_LSS         348 MEDENTDYSDLAPVNAAMNTVCCYV--RDGPGAYSVRRHVERLDEVLWVNAEGMLCNGTN 405
S.cerevisiae_LSS     328 TELQNTDSLCIAPVNQAFCALVTLI--EEGVDSEAFQRLQYRFKDALFHGPQGMTIMGTN 385

A.nidulans_LSS       400 AGHTWETAFVLQAYAEGGLSKVPEIQAAIERAYKYLVEQQHV-------------VDYPE 446
H.sapiens_LSS        383 GSQIWDTAFAIQALLEAGGHHRPEFSSCLQKAHEFLRLSQVP--------------DNPP 428
A.thaliana_CAS1      412 GSQLWDTGFAIQAILATNLVE--EYGPVLEKAHSFVKNSQVL-------------EDCPG 456
A.thaliana_CAS2      321 GSQLWDTGFAIQAILATNLVE--EYGPVLEKAHSFVKNSQVL-------------EDCPG 365
S.marinoi_CAS        836 GSQCWDTSFAIQAVWECGLLD--KFPILSAKVWAFLERTQILSTECSQSTPAYQYESC-D 892
C.criptica_CAS       359 GSQCWDTSFAIQAVRECGLLD--HFPLLSTKVWAYLERTQILSTETSQSSPAFQYESC-D 418
N.crassa_LSS         406 GVQCWDTAFAIQAIMDAGLTEDPRWRPMLIKSLEFLEDQQIR--------------ENVK 451
S.cerevisiae_LSS     386 GVQTWDCAFAIQYFFVAGLAERPEFYNTIVSAYKFLCHAQFD-------------TECVP 432

A.nidulans_LSS       447 DSQCHFFSRL--GGWPFSTRYQGNVCSDCTGEALKSILMIE-RDSRFT--------RLTT 495
H.sapiens_LSS        429 DYQ-KYYRQMRKGGFSFSTLDCGWIVSDCTAEALKAVLLLQEKCPHVT--------EHIP 479
A.thaliana_CAS1      457 DLN-YWYRHISKGAWPFSTADHGWPISDCTAEGLKAALLLS-KVPKEIVG------EPID 508
A.thaliana_CAS2      366 DLN-YWYRHISKGAWPFSTADHGWPISDCTAEGLKAALLLS-KVPKAIVG------EPID 417
S.marinoi_CAS        893 SRD-KFYRHVSKGGWPFSTSAHGWPISDCTGEGLKGVLALM-DSPIVMDGVKRGILKDIE 950
C.criptica_CAS       419 NRD-KFYRHVSKGGWPFSTSAHGWPISDCTGEGLKGVLALM-NSPIVVEAVKTGVLKSID 473
N.crassa_LSS         452 DQD-KCYRHPRKGAWAFSNKDQGYAVSDCVSEALKSVIILQ-KTPGFP--------TLID 501
S.cerevisiae_LSS     433 GS----YRDKRKGAWGFSTKTQGYTVADCTAEAIKAIIMVK-NSPVFSEV-----HHMIS 482
```

Supplementary Figure 6: Rooted phylogenetic tree of Sterol Methyl Transferases (SMTs) from various organisms. The bootstrapping test (replicate = 100) is indicated on each node in order to test the phylogeny. Nicotinate methyltransferase (NANMT) was used as outgroup. Red square = *S. marinoi*; Green square = *C. cryptica*; Light blue spot = other diatom.

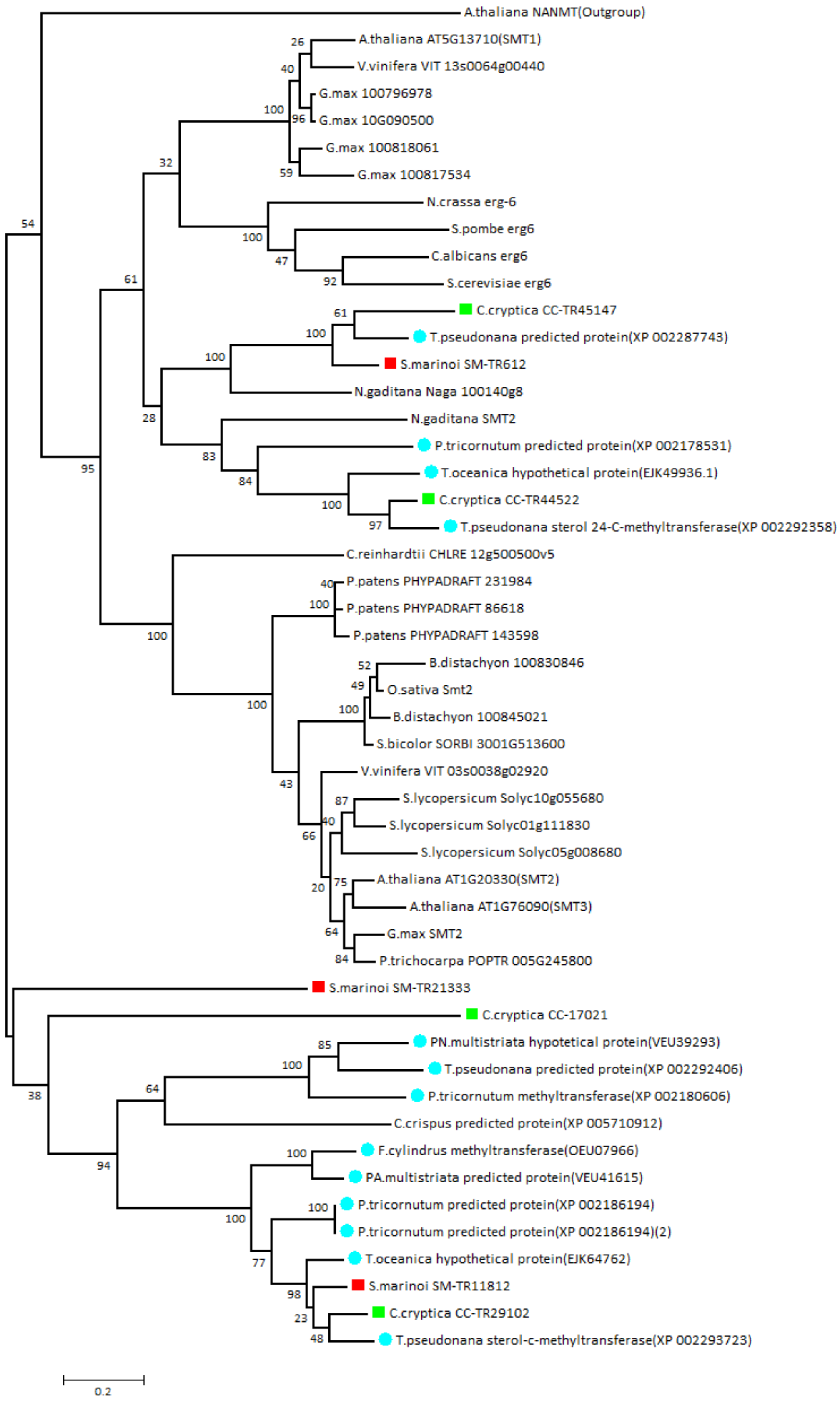

Supplementary Figure 7: Un-rooted phylogenetic trees of key enzymes of C5-sterol desaturase, including DWF7 and ERG3, from various organisms. The bootstrapping test (replicate = 100) is indicated on each node in order to test the phylogeny. Red square = *S. marinoi*; Green square = *C. cryptica*; Light blue spot = other diatom.

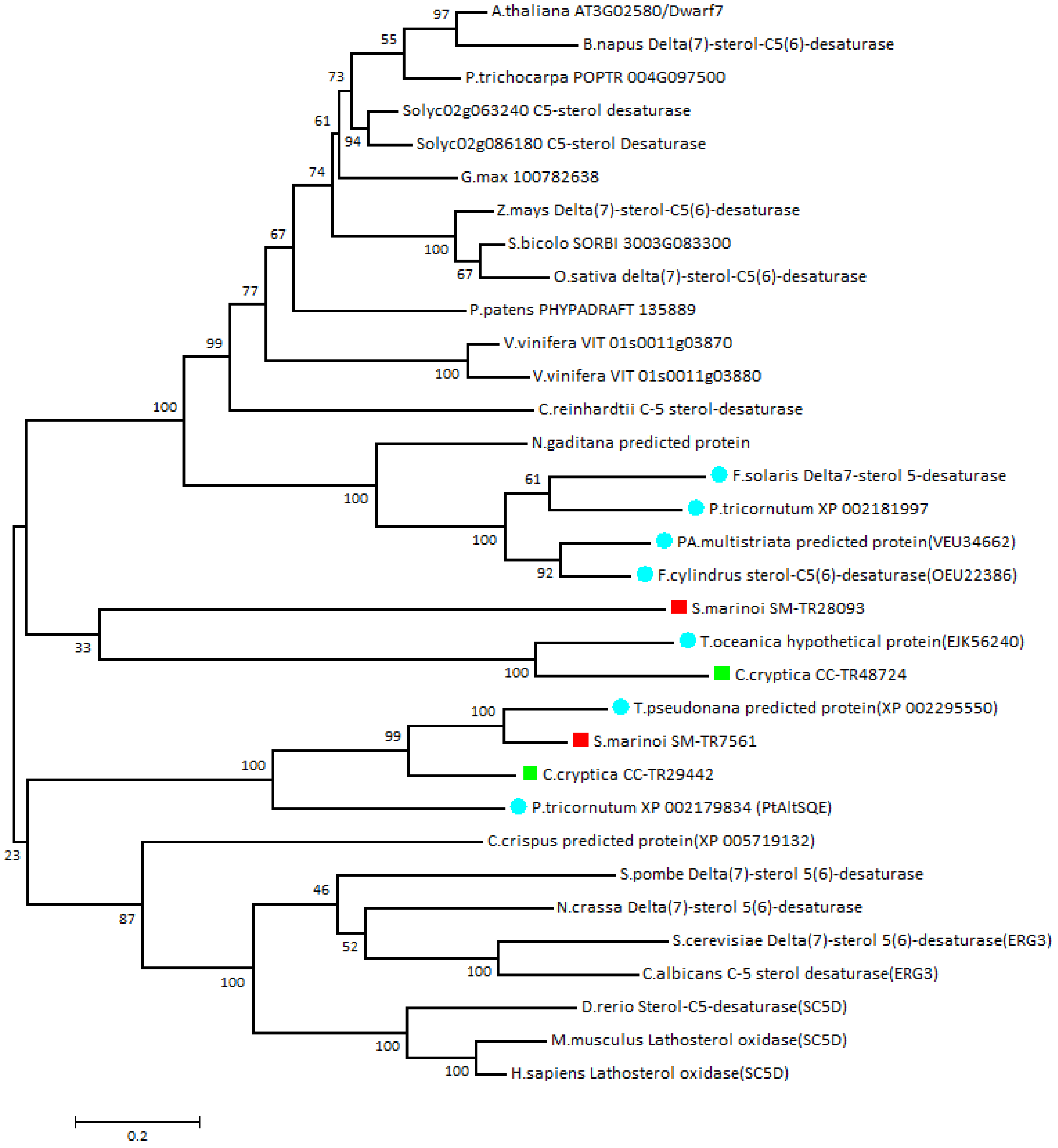

Supplementary Figure 8: Un-rooted phylogenetic trees of key enzymes of DWF5, ERG4 and 7 dehydrocholesterol reductase from various organisms. The bootstrapping test (replicate = 100) is indicated on each node in order to test the phylogeny. Red square = *S. marinoi*; Green square = *C. cryptica*; Light blue spot = other diatom.

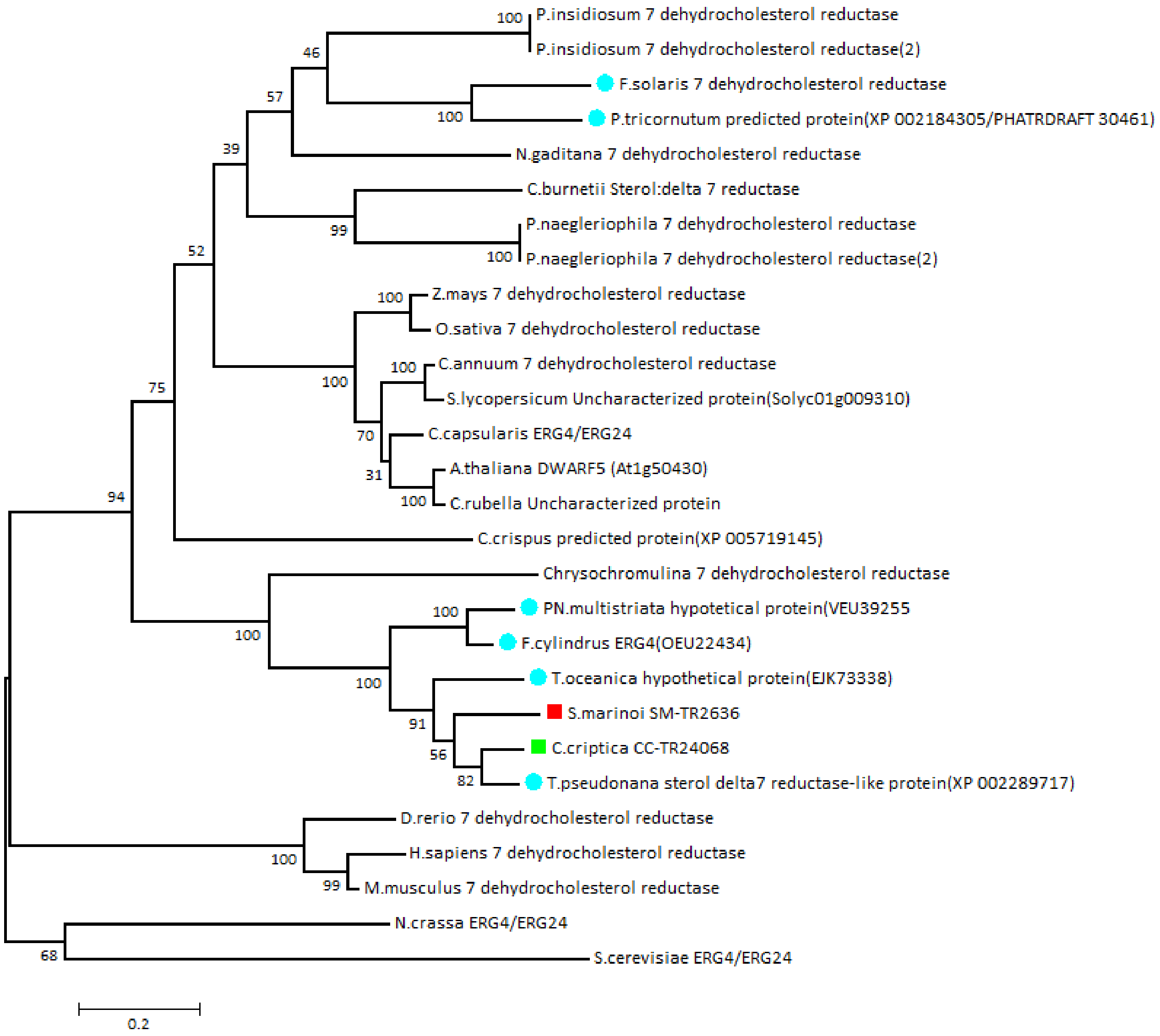

Supplementary Figure 9: Alignment of aminoacid sequences of DWF1 from *A. thaliana* and *S. lycopersicum*, SSR2 from *S. lycopersicum* and 24-DHCR from *Homo sapiens* and Sm-TR10998 from *S. marinoi*.

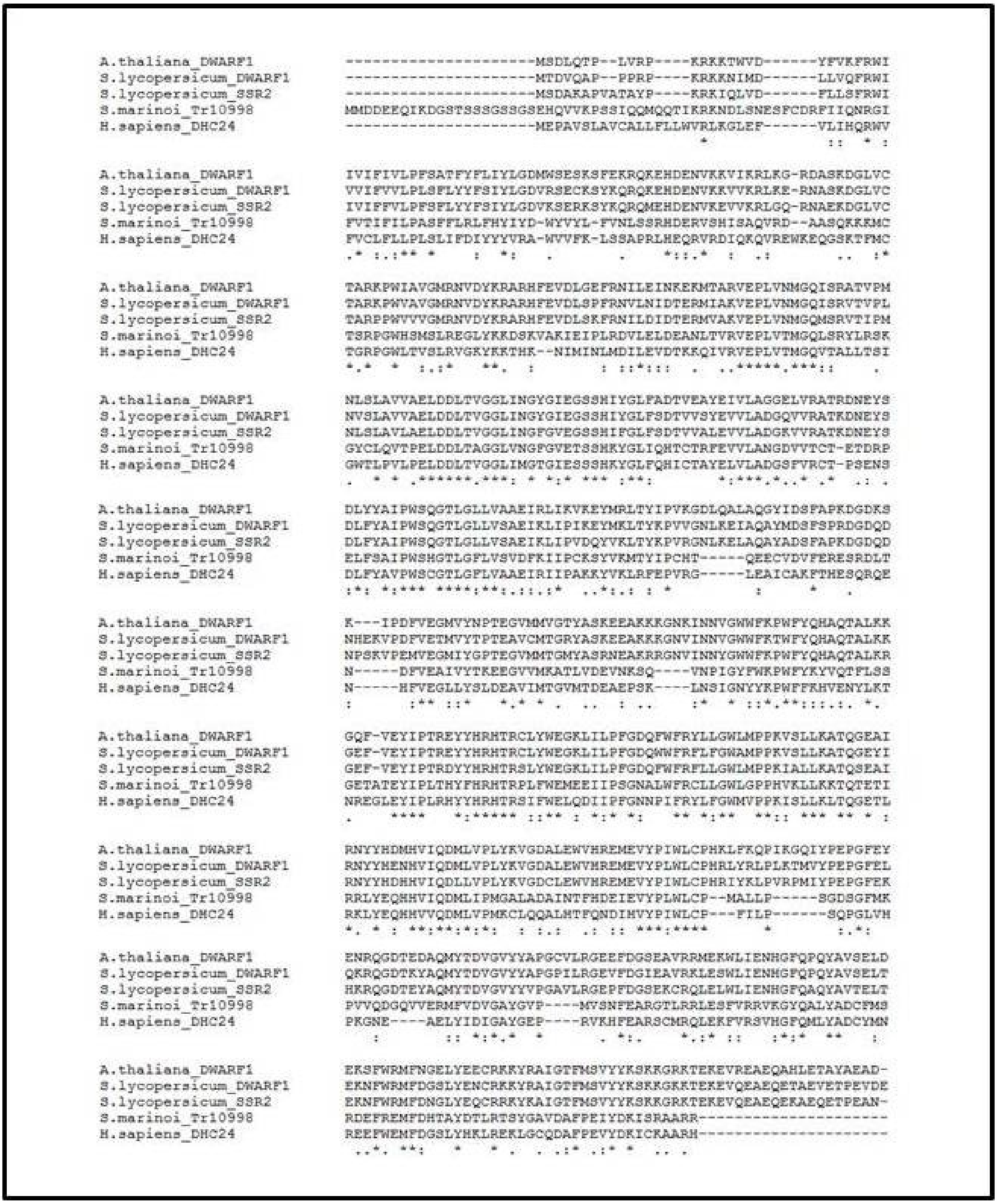

SUPPORTING MATERIAL

Diatoms synthesize sterols by inclusion of animal and fungal enzymes in the plant pathway

Carmela Gallo[1], Simone Landi[1,2], Giuliana d'Ippolito[1], Genoveffa Nuzzo[1], Emiliano Manzo[1], Angela Sardo[1,3], Angelo Fontana[1]

[1] National Research Council of Italy, Institute of Biomolecular Chemistry, Bio-Organic Chemistry Unit, Via Campi Flegrei 34, 80078 Pozzuoli (Napoli), Italy.

[2] Univeristy of Naples "Federico II", Department of Biology, Cupa Nuova Cintia 21 , 80126 Napoli, Italy

[3] Stazione Zoologica "A. Dohrn", Villa Comunale, 80121 Napoli, Italy

FIGURE SM1. ^{1}H NMR spectrum (600 MHz, C_6D_6) of acetylated 24-methylene cholesterol from *S. marinoi*.

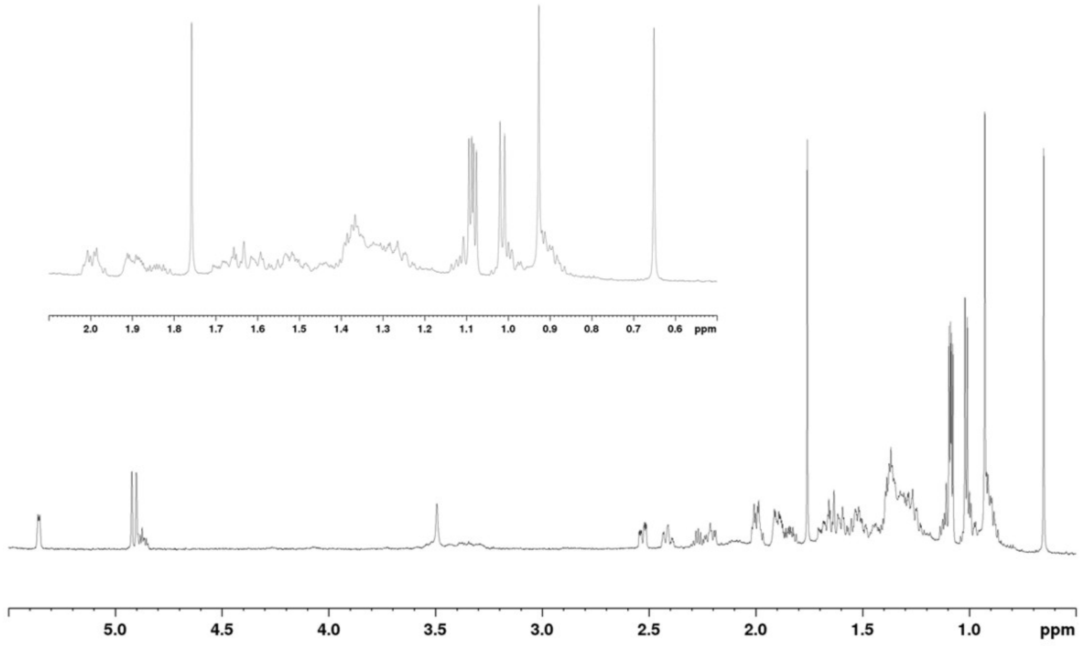

FIGURE SM2. ^{1}H NMR spectrum (600 MHz, $CDCl_3$) of natural dihydrobrassicasterol.

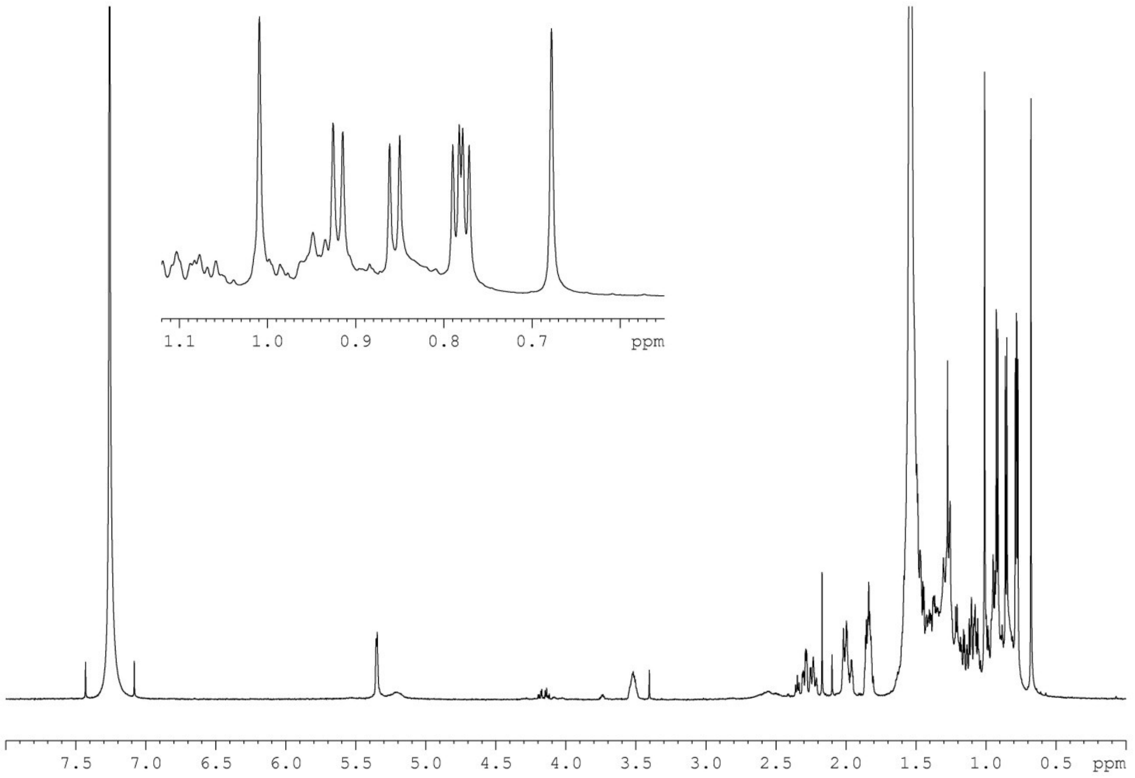

FIGURE SM3. ^{13}C NMR spectrum (125 MHz, $CDCl_3$) of natural dihydrobrassicasterol.

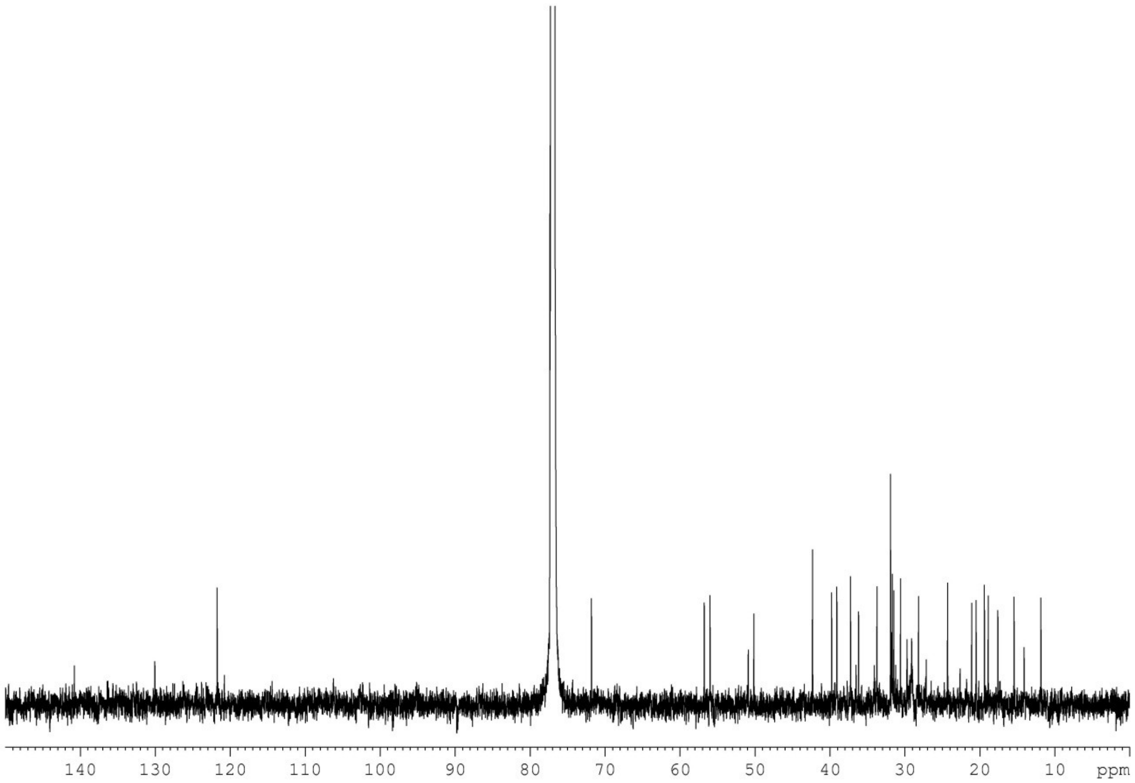

FIGURE SM4. ^{1}H NMR spectrum (600 MHz, $CDCl_3$) of clionasterol from *C. cryptica*.

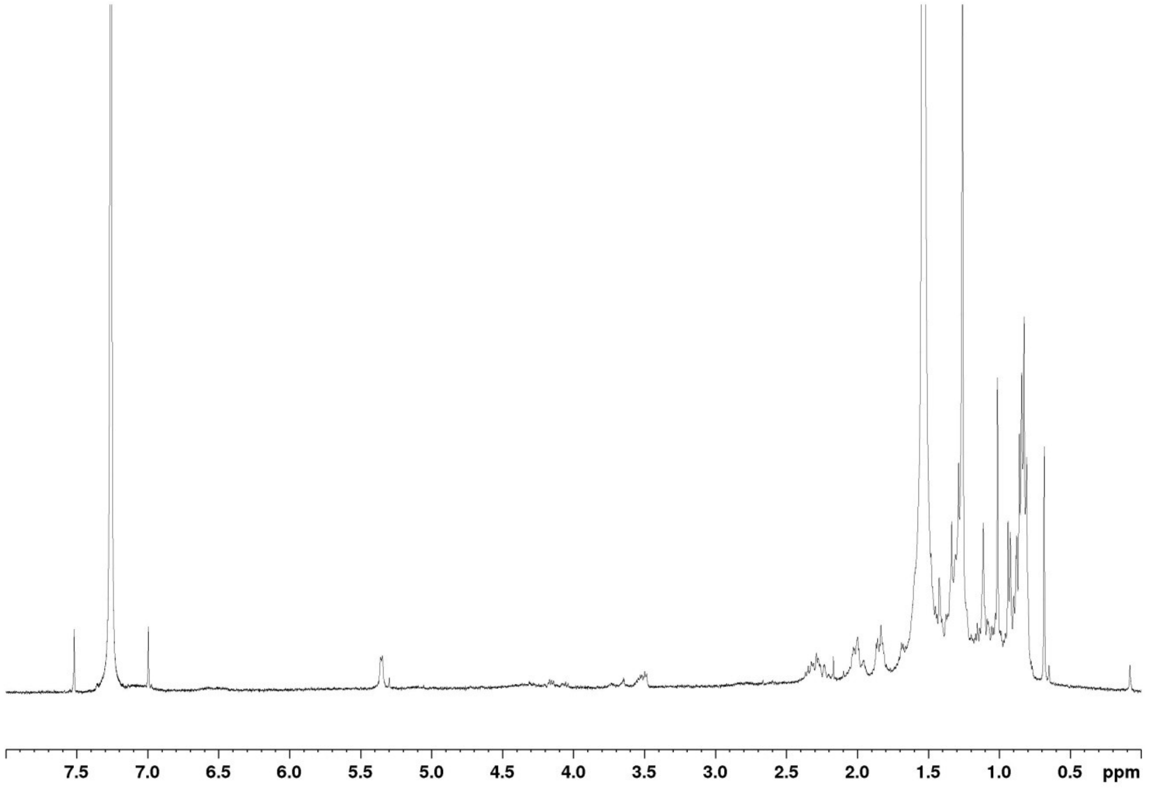

FIGURE SM5. COSY spectrum (600 MHz, $CDCl_3$) of clionasterol from *C. cryptica*.

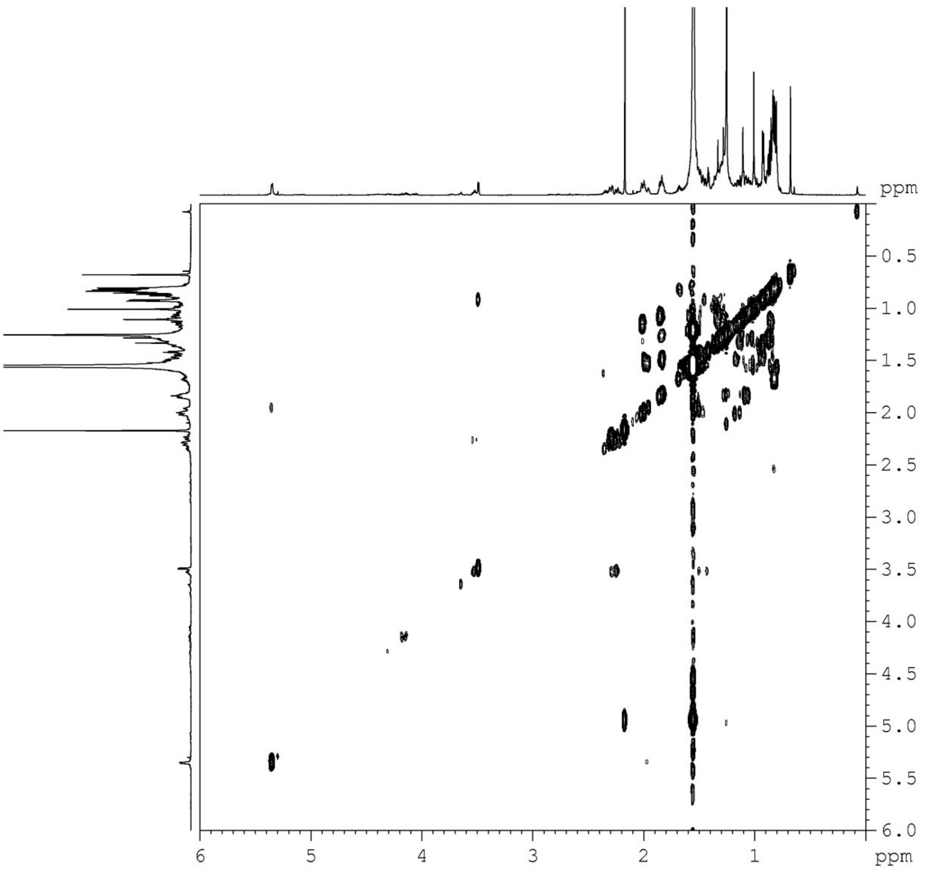

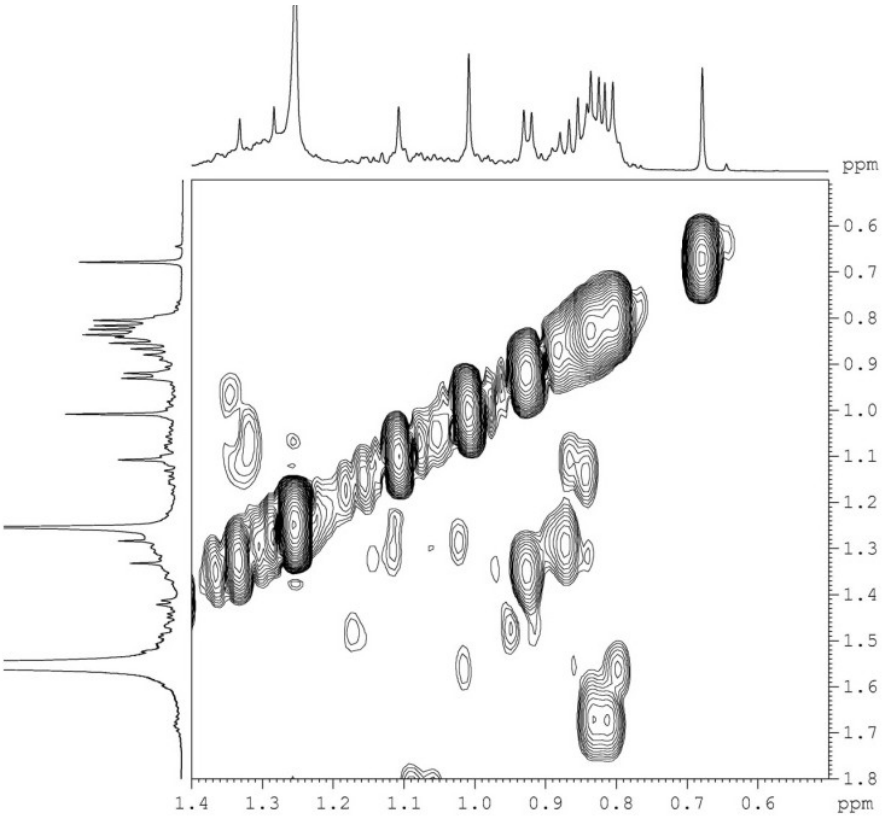
ppm
0.6
0.7
0.8
0.9
1.0
1.1
1.2
1.3
1.4
1.5
1.6
1.7
1.8
1.4 1.3 1.2 1.1 1.0 0.9 0.8 0.7 0.6
ppm

FIGURE SM6. HSQC spectrum (600 MHz, $CDCl_3$) of clionasterol from *C. cryptica*.

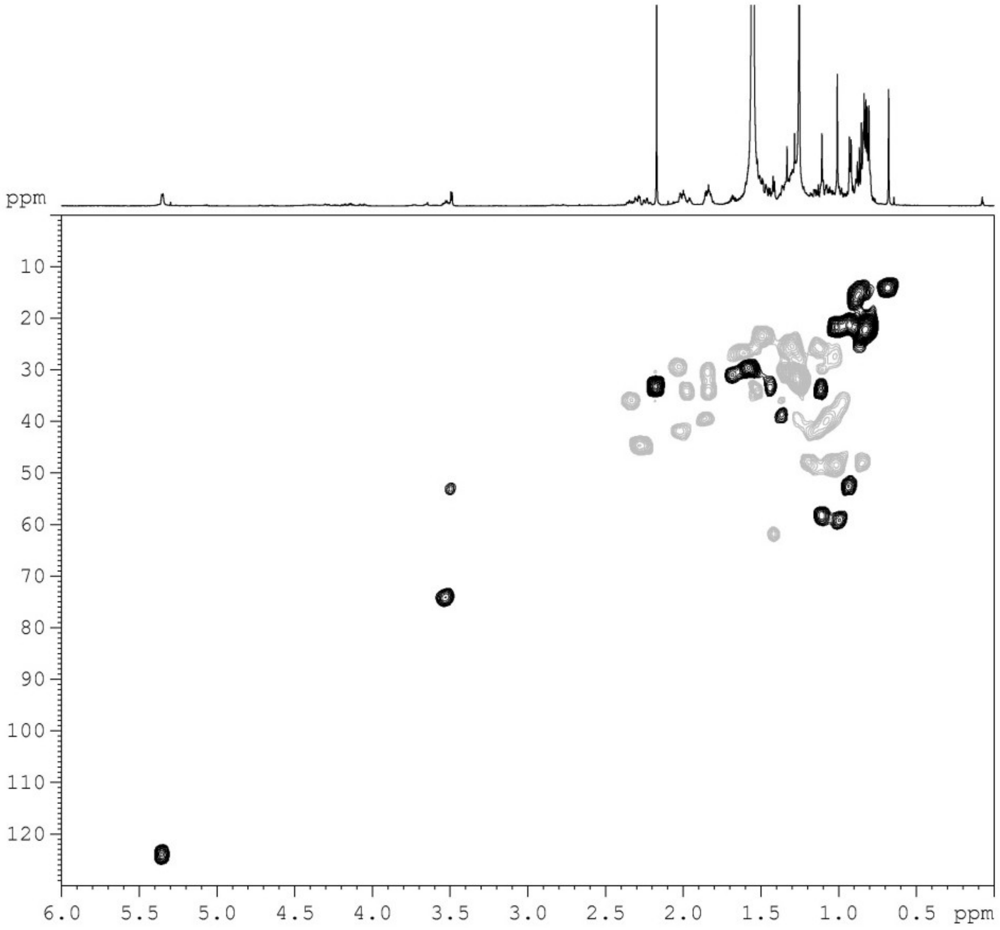

FIGURE SM7. ^{1}H NMR spectrum (600 MHz, C_6D_6) of natural fucosterol.

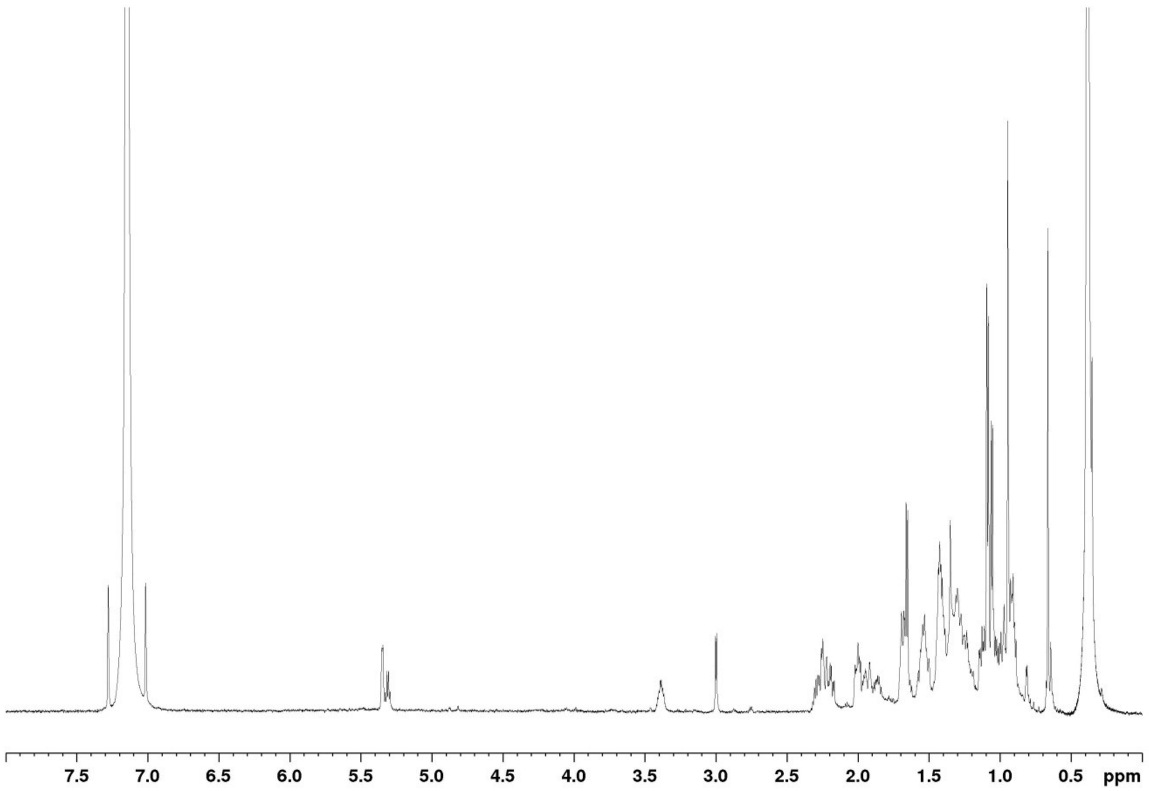

FIGURE SM8. ^{1}H NMR spectrum (600 MHz, $CDCl_3$) of acetyl brassicasterol from *P. tricornutum*.

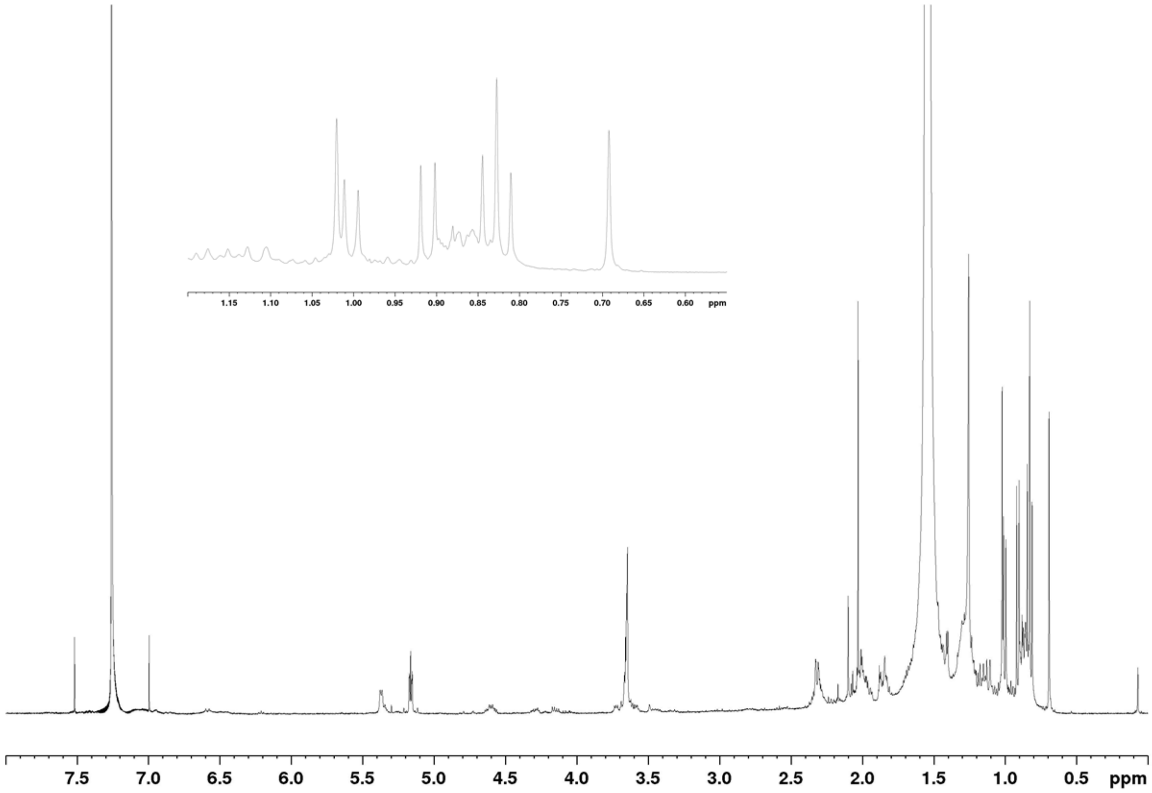

FIGURE SM9. COSY spectrum (600 MHz, $CDCl_3$) of acetyl brassicasterol from *P. tricornutum*.

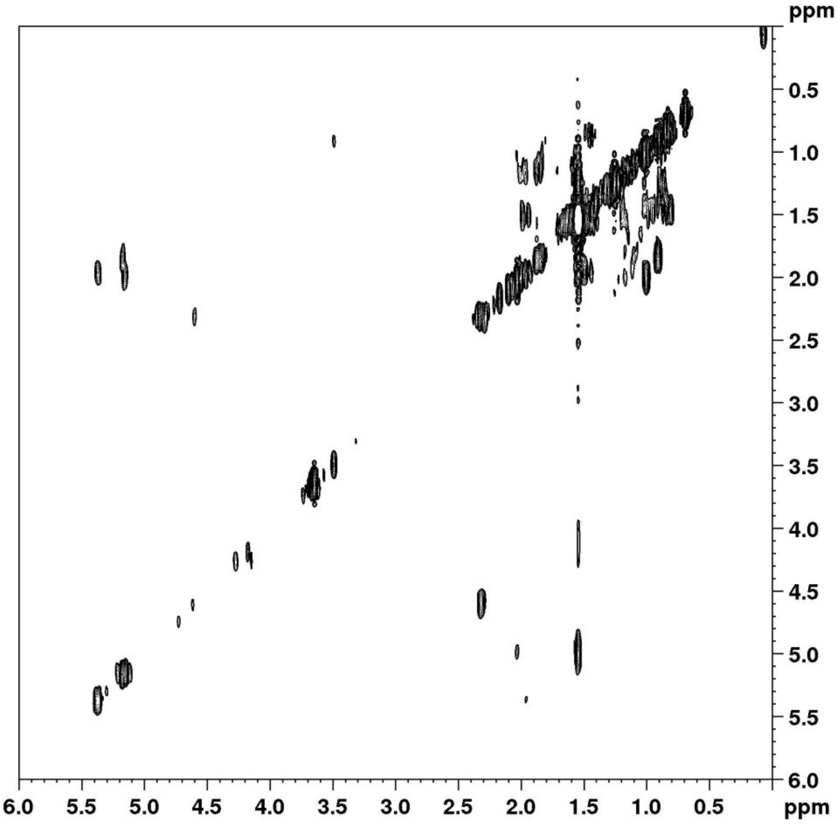

FIGURE SM10. Nodal sterol biosynthesis related translated transcripts comparison between *Skeletonema marinoi* (Queries) and *Cyclotella cryptica* transcriptome (Database).

- *Mevalonate Kinase: SM-MEVK (TR10489) obtains= CC-MEVK (TR35862)*

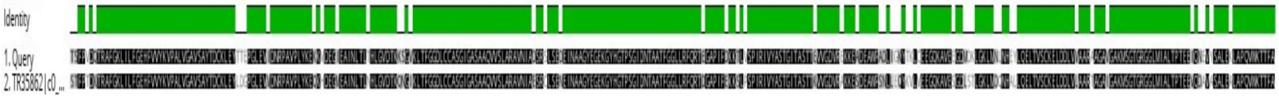

Query Coverage: 99.68%

Identities: 84.3%

Positives: 92.7%

- *Squalene synthase: SM-SQS (TR5837) obtains=CC-SQS (TR905)*

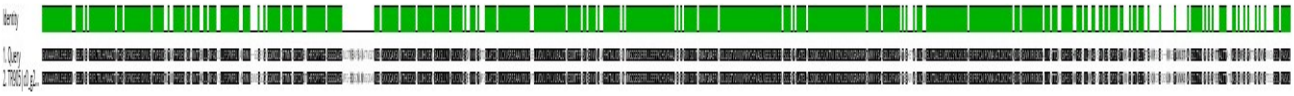

Query Coverage: 83.4%

Identities: 76.2%

Positives: 86.8%

- *Cycloartenol synthase: SM-CAS (TR12960) obtains=CC-CAS (TR3126)*

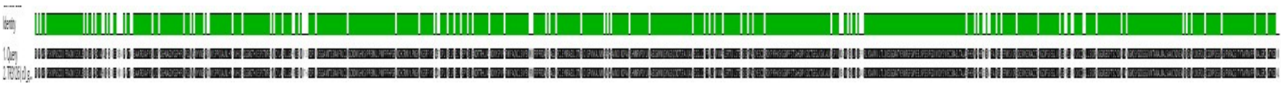

Query Coverage: 61.5%

Identities: 84.7%

Positives: 92.6%

Cycloartenol-C-24-methyltransferase: SM-SMT1 (TR612) obtains = CC-SMT1 (TR45147)

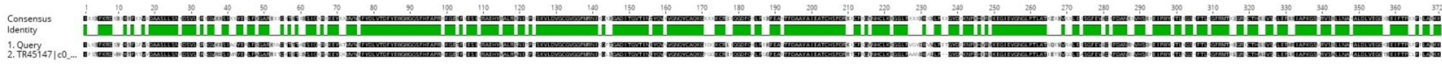

Query Coverage: 91.8%

Identities: 72%

Positivies: 82.3%

- *Sterol methyl transferase: SM-SMT2 (TR11812) obtains= CC-SMT2(TR29102)*

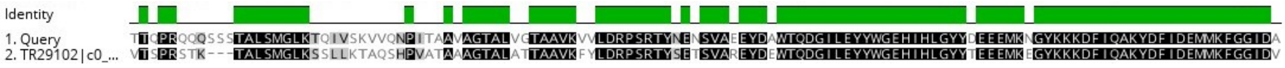

Query Coverage: 84.6%

Identities: 72.7%

Positivies: 78.5%

- *Sterol 14 demethylase: SM-CYP (TR7602) obtains= CC-CYP (TR23734)*

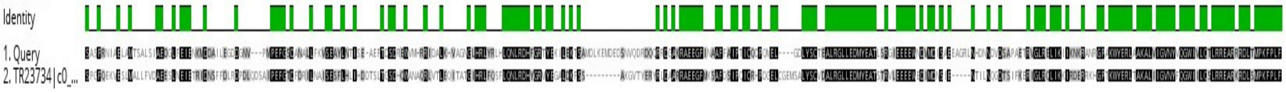

Query Coverage: 68.2%

Identities: 47%

Positivies: 64.8%

- *7 dehydrocholesterol reductase: SM-DWF5 (TR2636) obtains = CC-DWF5 (TR24068)*

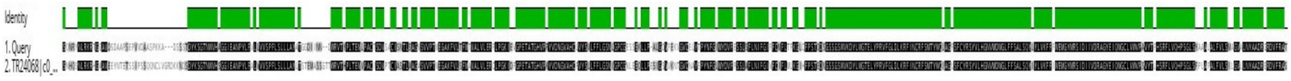

Query Coverage: 99.5%

Identities: 74%

Positivies: 83.7%

- *Delta(24(24(1)))-sterol reductase: SM-24SR (TR213) obtains= CC-24SR (TR29411)*

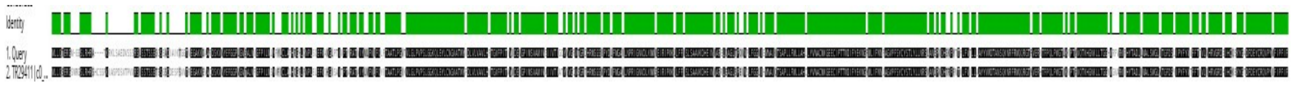

Query Coverage: 100%

Identities: 75.5%

Positivies: 85.2%

Alignments and outputs were performed using the Geneious software (Kearse et al., 2012). Blast investigation were performed using *S.marinoi* translated transcripts as queries; database was constructed using *C. cryptica* transcriptome. Reverse investigation were performed to confirm the results. This investigation revealed equal results (Data not showed).

Kearse M, Moir R, Wilson A, Stones-Havas S, Cheung M, Sturrock S et al. 2012. Geneious Basic: An integrated and extendable desktop software platform for the organization and analysis of sequence data. Bioinformatics 15;28 (12) 1647-9.

FiIGURE SM11. Nodal sterol biosynthesis related translated transcripts alignments between *Skeletonema marinoi* and *Solanum lycopersicum*

- *Cycloartenol synthase, SM-CAS (TR12960).*

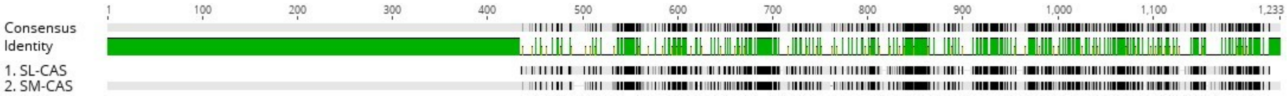

Identities: 43.7%

Positives: 58.2%

- *Cycloartenol-C-24-methyltransferase, SM-SMT1 (TR612)*

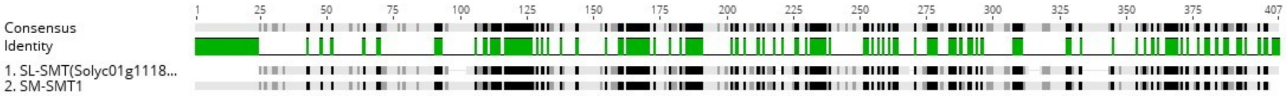

Identities: 32.4%

Positives: 50%

and

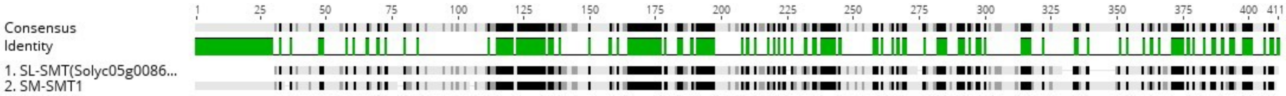

Identities: 34.2%

Positives: 50.4%

- *Sterol methyl transferase, SM-SMT2 (TR11812)*

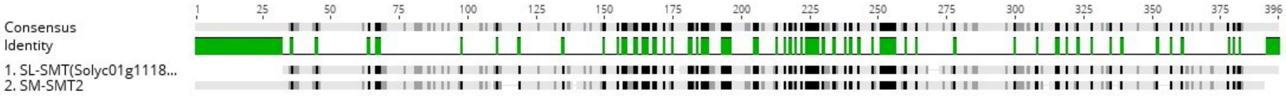

Identities: 20.6%

Positives: 39%

and

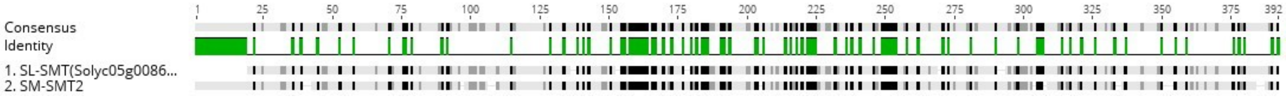

Identities: 23.3%

Positives: 39.7%

- *C-4 sterol methylsterol monooxygenase, SM-SMO(TR11964)*

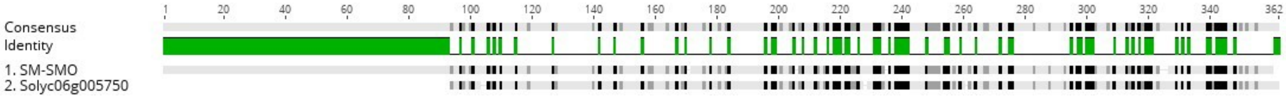

Identities: 25%

Positives: 41%

- *7 dehydrocholesterol reductase, SM-DWF5 (TR2636)*

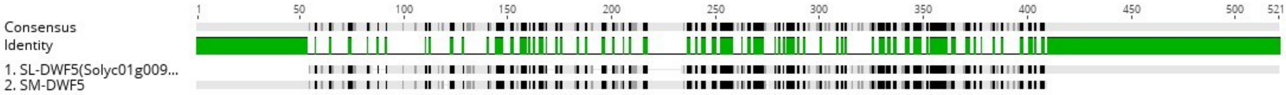

Identities: 31%

Positives: 47.3%

FIGURE SM12. Nodal sterol biosynthesis related translated transcripts alignments between *Cyclotella cryptica* and *Solanum lycopersicum*

- *Cycloartenol synthase, CC-CAS (TR3126).*

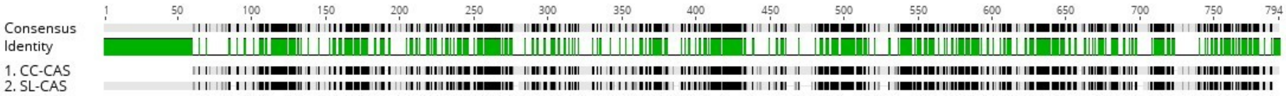

Identities: 46%

Positives: 60.8%

- *Cycloartenol-C-24-methyltransferase, CC-SMT1 (TR45147)*

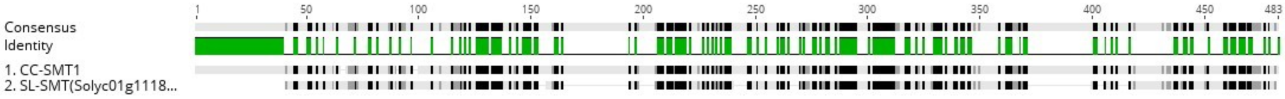

Identities: 33%

Positives: 45.2%

and

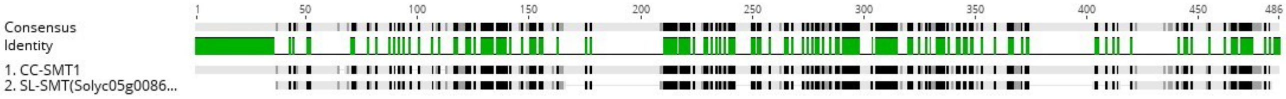

Identities: 33%

Positives: 45.9%

- *Sterol methyl transferase, CC-SMT2 (TR29102)*

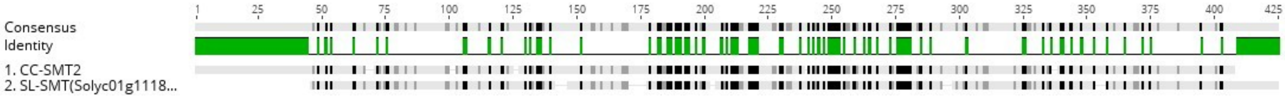

Identities: 21.8%

Positives: 36.9%

and

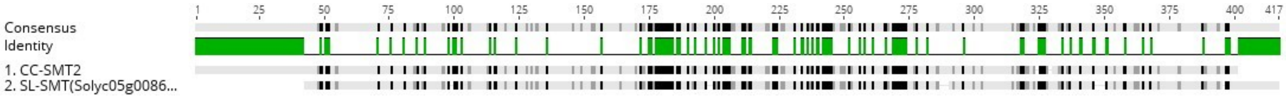

Identities: 22.6%

Positives: 37.6%

- *7 dehydrocholesterol reductase, CC-DWF5 (TR24068)*

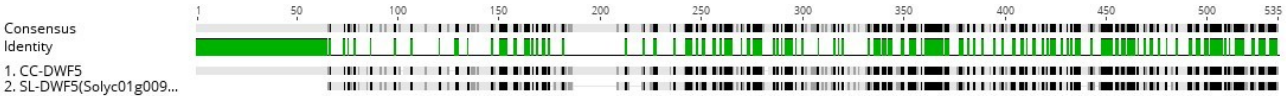

Identities: 35.5%

Positives: 52.6%

Alignments and outputs were performed using the Geneious software Alignments and outputs were performed using the Geneious software (Kearse et al., 2012)

Kearse M, Moir R, Wilson A, Stones-Havas S, Cheung M, Sturrock S et al. 2012. Geneious Basic: An integrated and extendable desktop software platform for the organization and analysis of sequence data. Bioinformatics 15;28 (12) 1647-9.
